# Decoding of the surfaceome and endocytome in primary glioblastoma cells identifies potential target antigens in the hypoxic tumor niche

**DOI:** 10.1186/s40478-024-01740-z

**Published:** 2024-02-27

**Authors:** Kelin Gonçalves de Oliveira, Anna Bång-Rudenstam, Sarah Beyer, Axel Boukredine, Hugo Talbot, Valeria Governa, Maria C. Johansson, Ann-Sofie Månsson, Karin Forsberg-Nilsson, Johan Bengzon, Johan Malmström, Charlotte Welinder, Mattias Belting

**Affiliations:** 1 Department of Clinical Sciences, Lund, Section of Oncology, Lund University, Barngatan 4, 221 85 Lund, Sweden; 2 Department of Immunology, Genetics and Pathology and Science for Life Laboratory, Uppsala University, Uppsala, Sweden; 3 Division of Cancer and Stem Cells, University of Nottingham Biodiscovery Institute, Nottingham, UK; 4 Department of Clinical Sciences, Section of Neurosurgery, Lund University, Lund, Sweden; 5 Department of Clinical Sciences, Division of Infection Medicine, Lund University, Lund, Sweden; 6 Department of Hematology, Oncology and Radiophysics, Skåne University Hospital, Lund, Sweden

**Keywords:** Immunotherapy, Glioblastoma, Tumor antigens, Proteomics, Hypoxia

## Abstract

Immunotherapies with antibody–drug-conjugates (ADC) and CAR-T cells, targeted at tumor surface antigens (surfaceome), currently revolutionize clinical oncology. However, target identification warrants a better understanding of the surfaceome and how it is modulated by the tumor microenvironment. Here, we decode the surfaceome and endocytome and its remodeling by hypoxic stress in glioblastoma (GBM), the most common and aggressive brain tumor in adults. We employed a comprehensive approach for global and dynamic profiling of the surfaceome and endocytosed (endocytome) proteins and their regulation by hypoxia in patient-derived GBM cultures. We found a heterogeneous surface-endocytome profile and a divergent response to hypoxia across GBM cultures. We provide a quantitative ranking of more than 600 surface resident and endocytosed proteins, and their regulation by hypoxia, serving as a resource to the cancer research community. As proof-of-concept, the established target antigen CD44 was identified as a commonly and abundantly expressed surface protein with high endocytic activity. Among hypoxia induced proteins, we reveal CXADR, CD47, CD81, BSG, and FXYD6 as potential targets of the stressed GBM niche. We could validate these findings by immunofluorescence analyses in patient tumors and by increased expression in the hypoxic core of GBM spheroids. Selected candidates were finally confronted by treatment studies, showing their high capacity for internalization and ADC delivery. Importantly, we highlight the limited correlation between transcriptomics and proteomics, emphasizing the critical role of membrane protein enrichment strategies and quantitative mass spectrometry. Our findings provide a comprehensive understanding of the surface-endocytome and its remodeling by hypoxia in GBM as a resource for exploration of targets for immunotherapeutic approaches in GBM.

**Supplementary Information:**

The online version contains supplementary material available at 10.1186/s40478-024-01740-z.

## Introduction

Glioblastoma (GBM) stands out as the most prevalent and aggressive primary malignant brain tumor in adults. Despite significant advances in our understanding of the underlying molecular drivers and biology of GBM, prognosis remains poor with a median overall survival of approximately 12–15 months [1]. The current therapeutic strategy, aiming at maximal safe surgical resection and radiochemotherapy, and in some cases tumor-treating fields, offers marginal survival benefit. Hence, the design of new treatment strategies for GBM is highly warranted.

Many factors pose challenges to the translation of research advances into novel therapeutic tools in GBM, including tumor heterogeneity, redundant signaling pathways, failure of target inhibition, the blood-tumor-barrier (BTB), and adaptive responses to microenvironmental stress [2, 3]. Hypoxia, a hallmark of GBM and one of the main tumor microenvironment stresses, arises once the tumor outgrows its vascular supply, triggering adaptive pathways, including angiogenesis and maintenance of stemness [4, 5]. Together, hypoxic adaptive mechanisms allow tumor cells to thrive within their hostile niche, develop resistance to conventional oncological treatments, and ultimately contribute to tumor relapse and poor survival.

Endocytosis represents the cellular “eat and drink” system that responds to hypoxic stress through spatial coordination of transport and signaling proteins in vesicular compartments [6–8]. Endocytic transport vesicles have come into great focus as targets for tumor-specific delivery of therapeutic substances, such as antibody–drug-conjugates (ADCs) and mRNA-loaded nanoparticles [9]. ADCs targeting *e.g.* HER-2 and > 10 additional tumor surfaceome antigens have been approved and implemented in the treatment of cancer, and several ADCs are currently tested in the clinic [10]. However, a significant bottleneck remains in the limited repertoire of identified tumor surface proteins and a lack of understanding regarding their endocytic capacity for ADC internalization. In this context, the hypoxic surfaceome emerges as a reservoir of potential targets for ADCs and adoptive immune cell therapy strategies, including CAR-T cells.

The identification of surfaceome targets has historically focused on differential mRNA expression between tumor and healthy tissues. However, several studies have indicated that mRNA constitutes a poor surrogate for actual protein availability [11, 12]. Hence, there is an increasing emphasis on proteomic approaches directed at the elusive plasma membrane proteome. Here, we applied a recently developed technique, tumor surfaceome mapping (TS-MAP) [13], to unravel the surface-endocytome makeup in GBM, and how it is reshaped by tumor mimicking hypoxic conditions. Our findings unveil a diverse selection of putative candidates that may be harnessed for targeted immunotherapies. Moreover, our integrated proteotranscriptomic approach provides compelling evidence that mRNA levels poorly reflect surfaceome-endocytome protein abundance in primary GBM cells, underscoring the importance of refined proteomic strategies.

## Materials and methods

### Patient derived specimens

Clinical specimens from six GBMs, three low-grade glioma (LGG) tumors, and three normal brain tissues from epileptic surgery were collected from patients referred to the Neurosurgery Department at Lund University Hospital, Sweden. Inclusion criteria were age 18 years or above, WHO performance status 0–2, and ability to give written informed consent. Patients were diagnosed by routine MRI of the brain, and surgical and pathological procedures, received standard oncological treatment and were followed up according to local and national recommendations.

### Cell cultures

Patient derived primary GBM cell cultures, U3034MG, U3047MG, and U3017MG, representative of different subtypes were obtained from the Human Glioma Cell Culture (HGCC, RRIDs: CVCL_IR73, CVCL_IR79, CVCL_IR63) non-commerical biobank resource at Uppsala University, Uppsala, Sweden, authenticated by short tandem repeat (STR) profiling [14]. The cells were seeded on dishes pre-coated with 10 µg/mL poly-L-ornithine hydrobromide (PLO, Sigma Aldrich, P3655) and 10 µg/mL laminin from Engelbreth-Holm-Swarm murine sarcoma basement membrane (Sigma Aldrich, L2020). Cells were routinely cultured in a 1:1 mix of Neurobasal (Gibco, 21103-049) and Dulbeco’s modified Eagle medium (DMEM) /F12 (Gibco, 31331-028) without serum, complemented with 1% (v/v) N2 supplement (Gibco, 17502-048), 2% (v/v) B27 (Gibco, 12587-010) supplement, 10 ng/mL human recombinant FGF2 (Peprotech, 100-18B), 10 ng/mL EGF (Peprotech, AF-100-15), 100 U/mL Penicillin and 100 µg/mL Streptomycin (PEST; Sigma Aldrich, P0781) (growth medium). Additionally, U87MG (ATCC, RRID: CVCL_0022) human high-grade glioma cells [15], authenticated by ATCC via STR, karyotyping, morphology, and cytochrome C oxidase I analysis, were routinely cultured in high-glucose DMEM (HyClone, Cytiva, SH30081.01) medium supplemented with 2 mmol/L L-glutamine (Sigma Aldrich, G7513), 100 U/ml Penicillin, 100 µg/ml Streptomycin, and with 10% (v/v) FBS (Sigma Aldrich, F7524) (full medium). Spheroid formation was done by seeding 0.5–3 × 10^4^ cells per well, according to cell size, in ultra-low attachment u-bottom plates (S-Bio, MS-9096UZ) containing growth medium and kept on an orbital shaker at 90 rpm, with medium exchanged on days 7 and 11. By day 14, spheroids displayed a clear hypoxic core, as observed by CA9 (M75 clone, BioScience Slovakia, AB1001) staining (for details, see Fluorescence Microscopy section). Cell detachment prior to seeding was performed with TrypLE Express Enzyme (Gibco, 12,605,010) for primary cells, or Trypsin/EDTA (Gibco, 25200056) for U87MG. All cells were grown in a humidified 21% O_2_ and 5% CO_2_ incubator at 37 °C and periodically tested for Mycoplasma via Hoechst 33342 (Life technologies, H1399) staining and high-resolution confocal microscopy. For experiments requiring hypoxia, cells were incubated in a humidified SCI-TIVE N–N Hypoxia workstation (Baker Ruskinn Technology) set at 5% CO_2_, 1% O_2_, and 37 °C for 24 h, or as indicated by the text.

### Biotinylation of surface and endocytosed proteins (TS-MAP)

To obtain surface and endocytosed proteins in normoxic and hypoxic cells, we employed the TS-MAP protocol [13]. Briefly, subconfluent primary cultures and U87MG cells pre-conditioned for 24 h hypoxia or normoxia were pre-incubated on ice for 10 min and maintained on ice during the following steps to prevent internalization. Cells were washed with PBS (with MgCl_2_ and CaCl_2_Mg/Ca-PBS) at pH 8 prior to incubation with a 1 mg/mL solution of membrane-impermeable biotin (EZ-Link Sulfo-N-hydroxysuccinimide-SS-biotin, Thermo Scientific, 21331) for 30 min, rinsed, incubated with 0.1 M glycine for 10 min to stop the reaction, and rinsed again prior to processing of surface biotinylated proteins (surfaceome). To capture endocytosed proteins (endocytome), biotinylated cells were put into endocytosis-permissive conditions (2 h at 37 °C under 21% or 1% O_2_) following incubation with sodium-2-mercaptoethanesulfonate (MesNa) (200 mM, 2 × 15 min) for removal of remaining cell-surface biotin. Next, endocytome samples were incubated with Iodacetamide (IAA) (5 mg/mL, 10 min) (Merk, I6125) and, for samples designated to proteomics profiling, additional blocking of free biotin was performed with unconjugated streptavidin incubation (15 min at 50 µg/mL; sigma, S4762) prior to cell lysis.

### High-affinity chromatography enrichment of biotinylated proteins

Biotinylated cells from hypoxic and normoxic conditions with and without endocytosis as described above were lysed for 20 min at 4 °C in RIPA buffer containing protease inhibitors. Lysates were clarified by centrifugation at 18,000 × g for 10 min at 4 °C. The collected soluble fraction was quantified with BCA Protein assay kit, diluted 1:4 with Mg/Ca–PBS supplemented with protease inhibitors, passed through a 0.45 µm SFCA syringe filter (Corning, CLS431220), and applied to a HiTrap streptavidin HP-1 ml column (GE Healthcare, 17-5112-01) pre-equilibrated in PBS 0.1% Triton X-100 (buffer A) using a peristaltic pump set at a flowrate of 250 µl/min to allow for binding of biotinylated proteins to the column’s gel matrix. The loaded column was transferred to an HPLC UPC 900 system (Amersham Biosciences) equipped with an online 280 nm UV detector and extensively washed, at a 1 ml/min flow, with 10 mL of buffer A, followed by 10 mL wash with buffer B (50% buffer A, 50% RIPA buffer (v/v), 1 M NaCl), and finally 10 mL of buffer A to remove non-biotinylated proteins. For elution of proteins, the HPLC system was equilibrated with elution buffer (freshly prepared 150 mM MesNa in PBS 0.1% Triton X-100) and biotinylated proteins were released from the column by reduction of the disulfide link between protein and biotin moiety, at a flowrate of 125 µL/min. When the eluate reached 10 mL, one volume of 20% trichloroacetic acid (TCA, Sigma, T6399) in dH_2_O was added, homogenized with the protein solution, and incubated on ice for 30 min. Then, eluted proteins were pelleted by centrifugation (10 min at 18,000 × g at 4 °C), washed twice with 2% sodium acetate (Merck, #1.06264, w/v), resuspended in 30–70 µL (according to pellet size) of 6 M urea in 50 mM ammonium bicarbonate (AMBIC, Merck, #09830) buffer, aliquoted for protein quantification and stored at − 80 °C until LC–MS/MS sample preparation.

### Sample preparation for LC–MS/MS

Enriched protein solutions were further diluted in urea 6 M buffer (5 × the sample volume), homogenized, and kept at room temperature (RT) for 1 h. Samples were reduced with 10 mM dithiothreitol (Fisher Scientific, R0861) for 1 h at 56 °C while stirring (350 rpm) and alkylated with 50 mM IAA for 30 min in the dark, followed by buffer exchange with AMBIC solution pH 8.0. Protein samples were digested O/N with 0.4 µg/µL sequencing grade trypsin (Promega, V5111) in AMBIC solution (37 °C, 350 rpm). Trypsinization was stopped by adding 1% Trifluoroacetic acid (TFA, Merck, T6508) and samples were dried with SpeedVac Plus Centrifuge (Savant, SC110A). Samples were resuspended in 100 µl of 0.1% TFA in dH20 and desalted as per the manufacturer’s instructions with Ultra MicroSpin silica C18 columns (The Nest Group, SUM SS18V). Eluted desalted peptides were dried with SpeedVac and kept at − 20 °C short term. Prior to LC–MS/MS analysis, samples were resuspended in 10 µL of a 2% Acetonitrile (ACN, Göteborgs Termometerfabrik, 60–102930) and 0.1% TFA in dH_2_O solution, quantified by DS-11 spectrophotometer (DeNovix, DS-11), adjusted to final concentration of 0.5 µg/µL, centrifuged at 14,000 × g for 5 min and transferred into mass-spectrometer vials, which were stored at − 20 °C until being loaded into the instrument.

### Mass spectrometry acquisition

Peptides from GBM cultures were detected on a Tribrid mass spectrometer Fusion (Thermo Scientific). The equipment, coupled with a Nanospray Flex as ion source and with an EASY-nLC 1000 ultrahigh pressure liquid chromatography (UHPLC) pump (Thermo Fischer Scientific), received injections of 1 µg of peptides each in three independent experiments. Peptides were first concentrated on an Acclaim PepMap 100 C18 precolumn (75 µm × 2 cm, Thermo Scientific, Waltham, MA) and then separated on an Acclaim PepMap RSLC column (75 µm × 25 cm, nanoViper, C18, 2 µm, 100 Å) at 40–45 °C and flowrate of 300 nL/min. Solvent mix was used to generate a nonlinear peptide elution gradient (Solvent A: 0.1% formic acid in water, and solvent B: 0.1% formic acid in acetonitrile). Solvent B percentage plan was: 3% for 3–5 min; 20–30% for 90 min; then, gradient was either put at 30% for 20 min or increased to 60% for 10–15 min; increased to 90% for 5 min; and lastly, kept at 90% for 5–7 min for column cleansing. Acquisition mode was positive data-dependent acquisition (DDA). Peptides were introduced into the instrument via stainless steel Nano-bore emitter (OD 150 µm, ID 30 µm) with spray voltage at 2 kV and capillary temperature at 275 °C. The Orbitrap detector performed full MS survey scans (from m/z range of 350 to 1350) with resolution of 120,000. Automatic gain control (AGC) target was set to 4 × 10^5^ with injection time of 50 ms. Up to 20 most intense ions from the full scan MS with charge states 2–5 were selected for fragmentation in the Orbitrap, precursors (MS2) were isolated on the quadrupole mass filter set to 1.2 m/z of width and underwent high-energy collision dissociation (HCD) fragmentation at 30% of normalized collision energy (NCE). Resolution was fixed at 30,000 and for MS/MS scans, AGC target value and injection time were 5 × 10^4^ and 54 ms, respectively. Dynamic exclusion duration was set to 45 s and the mass tolerance window was 10 ppm.

Raw DDA data was analyzed with Proteome Discoverer™ 2.3 (PD 2.3) Software (Thermo Fisher Scientific), in which the peptides were identified with SEQUEST HT paired with UniProtKB human database (release 2020_05). Peptide search was performed with cysteine carbamidomethylation as a static modification, and N-terminal acetylation as well as methionine oxidation as dynamic modifications. Precursor tolerance was 10 ppm, fragment tolerance was set to 0.05 Da, up to 2 missed cleavages were allowed, and peptide validation was done with Percolator with maximum q-value of 0.05.

### Total RNA extraction, sequencing, and data processing

Primary cells seeded into T25 flasks (0.3–0.5 × 10^6^ cells/flask) and left to attach overnight (O/N) were pre-conditioned to 24 h normoxia/hypoxia (biological triplicates), detached with StemPro Accutase Cell Dissociation Reagent (Gibco, A1110501), collected in medium, and centrifuged for 5 min at 300 × g. The pellets were resuspended in PBS (Cytiva, SH30028.02) for cell counting, centrifuged for 5 min at 300 × g and each sample was resuspended in 350 µL RLT lysis buffer provided by the AllPrep DNA/RNA Mini Kit (Qiagen, 80,204) supplemented with 1% (v/v) β-mercaptoethanol (Sigma, M3148). Total RNA/DNA was then extracted as per manufacturer’s instructions. Nucleotide concentration and purity were determined with NanoDrop (ND1000) and further confirmed with Agilent Bioanalyzer QC at the Bioinformatics and Expression Analysis (BEA) core facility (Karolinska Institute, Stockholm), where RNAseq was performed. Clustering was done by 'cBot' and samples were sequenced on NovaSeq6000 (NovaSeq Control Software 1.6.0/RTA v3.4.4) with a 2 × 151 setup using 'NovaSeqXp' workflow in 'S4' mode flowcell. Raw sequencing data was demultiplexed and converted to FastQ. The Bcl to FastQ conversion was performed using bcl2fastq_v2.20.0.422 from the CASAVA software suite. The quality scale used was Sanger / phred33 / Illumina 1.8 + . Data processing prior to analysis started with paired-end adapter trimming performed with Trim Galore (version 0.6.4_dev), alignment with Human reference genome (Homo sapiens, GRCh38) using STAR software (version 2.7.3a), reads quality control checks with fastQC (version 0.11.9), and reads quantification as counts with featureCounts (version 2.0.0). Output and quality control report was generated with multiQC (version 1.8). Background correction, normalization, and differential gene expression analysis of protein-coding genes were performed with the DESeq2 r package (version 1.38.6) followed by gene annotation performed with biomaRt r package. For profiling of U87MG mRNA, cells were grown in serum-free DMEM and pre-conditioned to 24 h normoxia or hypoxia in triplicates. Total RNA was extracted with RNeasy mini kit (Qiagen, 74,104), quality control (Bioanalyzer) and processing were performed at the SCIBLU Genomics Centre at Lund University for hybridization on HumanHT-12v4 Expression Illumina BeadChip. Downstream background correction, normalization, and differential expression analysis were performed with limma r package followed by gene annotation with illuminaHumanv4.db r package.

### Enrichment analysis of RNAseq and LC–MS/MS data

For subtyping of normoxic and hypoxic samples, Z-scored gene expression of primary GBM and U87MG cells were used as input for Preranked Geneset Enrichment Analysis (GSEA, v4.2.3, Broad Institute [16, 17] analysis with Verhaak et al*.* GBM subtype signatures [18]; available at MSigDB) as genesets and default software settings. For proteomics data, enrichment of surface proteins in biotinylated samples (Surface and Endocytosis fractions) was verified by comparing identified surface/endocytosed protein identities to proteins identified in MesNa-treated cells. For this, grouped protein abundances obtained after proteomics data processing for all involved samples were used as GSEA input matrix. SURFME list [13] was used as “geneset”, with Ratio of Classes as metric parameter, gene_set as permutation parameter, set_max at 4000, and default remaining settings. For GSEA of hypoxic vs. normoxic profile, log_2_ gene expression values (output from DEseq2 rlog function) of all protein-coding genes or log_2_ protein abundances after proteomics data processing were used as input. The GSEA was run with HALLMARK_HYPOXIA as geneset (available at MsigDB) with difference of classes as metric parameter, gene_set as permutation parameter, and default remaining settings.

### Western blot

Normoxic or hypoxic cells were lysed with radioimmunoprecipitation assay (RIPA) buffer (10 mM Tris–HCl pH 7.4, 150 mM NaCl, 1 mM EDTA, 0.1% SDS, 1% Triton X-100, 1% sodium deoxycholate) supplemented with Protease Inhibitor Cocktail (Complete, 04693116001, Roche) for 20 min at 4 °C, followed by lysate homogenization. Cell lysates were clarified by centrifugation at 14,000 × g for 10 min at 4 °C, soluble supernatant was kept, quantified by BCA Protein Assay Kit (Pierce, 23,225), and stored at − 80 °C until used in further analysis. For HIF1α western blots, equal protein amounts (30 µg) were mixed with 4X NuPAGE LDS Sample Buffer (Invitrogen, NP0007) with reducing agent, heated for 10 min at 80 °C and loaded to NuPAGE 4–12% Bis–Tris gels (Invitrogen, NP0321PK2) for electrophoretic separation (200 V for 1 h) with SeeBlue Plus2 Prestained Ladder (Invitrogen, LC5925) as molecular mass standard. Samples were then transferred to polyvinylidene difloride membranes (Immobilon-FL, PVDF; Merck Millipore, IPFL00010) on a wet electroblotting system (30 V and 1.5 h), following blocking with 5% (w/v) skim milk (Sigma-Aldrich, 70,166) in TTBS for 1 h at RT and O/N incubation with primary antibodies at 4 °C on 90 rpm. Membranes were subsequently washed with Tris-buffered Saline with 0.5% (v/v) Tween 20 (TTBS, Fisher bioreagents, BP337), incubated with horseradish peroxidase-conjugated (HRP) secondary antibodies for 1 h at RT, washed and incubated for 1 min with enhanced chemiluminescence (ECL) substrate (Pierce, Thermo Scientific, 32,209). Blots were revealed with Odyssey XF Imaging System (LI-COR Biosciences). The antibodies used were: anti-HIF1α (GeneTex; GTX127309, RRID: AB_2616089 at 1:1000), anti-β-actin (Abcam; ab8227, RRID: AB_2305186 at 1:10,000), and anti-rabbit HRP (Cell Signaling Technology; #7074, RRID: AB_2099233 at 1:3000). For biotinylated samples, cells first underwent the TS-MAP protocol (described above). Biotinylated surface proteins, biotinylated endocytosed proteins, and MesNa (Thermo Scientific, M1511) treated control samples (5 µg, 10 µg, and 10 µg of protein, respectively) were prepared at non-reducing conditions and separated in a NuPAGE 4–12% Bis–Tris gel, under the same ladder and settings as described above. Electroblotting to PVDF membrane and blocking also occurred as previously described, following membrane incubation with Streptavidin HRP-linked (Thermo Scientific, N100, 1:2000) in TBST containing 3% bovine serum albumin (BSA, Sigma-Aldrich, A4503) for 1 h at RT and subsequent ECL substrate addition. Membranes were developed with chemiluminescence-reactive film.

### Fluorescence-activated cell sorting (FACS)

For quantification of global biotinylated surfaceome and endocytome (1 h endocytosis), cells underwent gentle detachment with TrypLE solution at 37 °C for 5 min, and were then collected in cold full medium, washed with ice-cold PBS, fixed for 10 min with 2% paraformaldehyde (PFA, BDH, 294474L), and rinsed with PBS. Endocytosis samples were permeabilized for 30 min with 0.5% saponin (Fluka BioChemika, 47,036) in PBS at RT. After washing, all samples underwent blocking with 3% BSA in PBS for 30 min at RT before labeling with 5 µg/mL Streptavidin-Alexa Fluor (AF)-488 (S32354, Life Technologies) in 3% BSA-PBS for 30 min at RT, rinsed and kept in cold PBS until acquisition. Data was acquired on Accuri C6 flow cytometer equipped with a 488 nm wavelength excitation laser and FL1-H detector (533/30 nm) and analyzed using Accuri C6 software (BD Biosciences). Results were expressed as mean fluorescence intensity (MFI) after subtraction of the values of negative control (cells only) and, to express the endocytosed protein–biotin fraction, values for this group underwent subtraction of residual biotin signal by MesNa treatment.

### Fluorescence microscopy

For visualization of biotinylated proteins, primary GBM cells were seeded (2–3.5 × 10^4^ cells/well) into 8-well chambers slides (Ibidi, 80,827), left O/N to adhere at 37 °C, pre-conditioned to normoxia or hypoxia for 24 h, treated according to the TS-MAP protocol (surface, 1 h endocytosis, MesNa-treated, and untreated controls), and fixed with PFA 4% at RT for 10 min. Biotinylated samples submitted to endocytosis were permeabilized with 0.5% saponin (Fluka BioChemika, 47,036) in PBS for 30 min at RT. Nonspecific sites were blocked with PBS containing 5% goat serum (Dako, X0907) for 1 h at RT, and cells were labeled with 20 µg/ml Streptavidin-AF-488 (Thermo Fisher, S32354) in 5% goat serum PBS for 30 min at RT. For surface staining, cells were seeded into chamber slides, left O/N to adhere at 37 °C, pre-conditioned to normoxia or hypoxia for 24 h, washed on ice, PFA fixed, blocked with 5% goat serum, incubated with 5 µg/mL of corresponding primary antibodies in 5% goat serum in PBS at 4 °C O/N, following 1 h RT incubation with respective anti-mouse or anti-rabbit AF-488 conjugated secondary antibody (5 µg/mL) in 5% goat serum in PBS. For visualization in 3D cultures, spheroids grown for 2 weeks were harvested, fixed with PFA 4% for 15 min, incubated O/N in 0.5 M sucrose (Sigma-Aldrich, S0389) at 4 °C, embedded in optimal cutting temperature (OCT) medium (Fisher Scientific, 12,678,646) and cryosectioned into 12 µm (primary GBM) or 6 µm (U87MG) sections. Sections were washed in PBS, blocked at RT with goat serum 5% in PBS for 1 h followed by O/N primary antibody (5 µg/mL) incubation at 4 °C, and incubated with anti-mouse or anti-rabbit AF-488-conjugated secondary antibody in the dark for 1 h. Lastly, for stainings of human tissues, 6 µm cryosections were cut from isopentane snap frozen tissue specimens, then rehydrated in PBS for 10 min, PFA-fixed and blocked as described above, followed by O/N incubation with primary antibody at 4 °C in a humidified chamber. Slides were washed and incubated with respective AF-conjugated secondary antibody for 1 h at RT. Nuclei were counterstained with Hoechst 33,342 (1/10000) for 10 min. Spheroid and tissue sections were then mounted with fluorescent mounting medium (Dako, S3023) prior to image acquisition. Primary antibodies used were: mouse monoclonal anti-CD44 (Abcam, ab264546), rabbit polyclonal anti-SLC2A3 (Sigma, HPA006539), rabbit monoclonal PE anti-CA9 (Abcam, ab275578), mouse monoclonal anti-CA9 (M75), rabbit monoclonal anti-CXADR (Invitrogen, MA5-29,208), mouse monoclonal anti-CD47 (Abcam, ab213079), rabbit monoclonal anti-CD81 (Abcam, ab219209), rabbit monoclonal anti-FXYD6 (Abcam, ab181254), and mouse monoclonal anti-BSG (Abcam, ab666). Secondary antibodies used were: goat polyclonal anti-mouse IgG-AF488, and goat polyclonal anti-rabbit IgG-AF488 (Thermo Fisher Scientific A-11001, A-11008).

Stained spheroid and patient tissue sections were captured on a Zeiss AxioScan Z1 slide scanner equipped with LED modules 385 and 475 nm, and a Plan-Apochromat 20 × /0.80 M27 objective. Imaging of biotinylated samples, 2D stainings, and details of tumor and spheroid sections was performed using Zeiss LSM 710 or 980 Axio Observer confocal scanning microscope equipped with excitation laser wavelengths of 405 and 488 nm, and Plan-Apochromat 63 × /1.40 DIC M27 or Plan-Neofluar 40x/1.30 Oil DIC M27 oil immersion objective, integrated with Zen software (Carl Zeiss).

### Incucyte live cell assays

All live assay imaging was performed using an Incucyte® S3 system placed in a humidified 5% CO_2_ incubator at 37 °C. Analysis was performed with Incucyte S3 integrated software. Primary cells or U87MG cells were seeded in 96-well plates (Thermo Scientific, 167,008) (pre-coated with laminin for primary cells) at a density of 2.5–7.5 × 10^3^ cells per well, left to attach O/N and, when required, pre-conditioned to normoxia/hypoxia 24 h before the start of the assay. For cell size assessment, the Incucyte Cell-by-Cell Analysis Software Module was used. After image acquisition, individual cells were defined by cell-by-cell segmentation, and the average phase area per cell was calculated for each primary cell line. Cell size is expressed as Phase area object average (μm^2^) ± S.D, from one representative measurement including > 8 wells per cell line. For antibody internalization experiments, anti-CD44 antibody or untargeted primary antibody (mouse isotype control IgG; Invitrogen, 31,903) was pre-complexed with Incucyte® Mouse IgG1 FabFluor-pH Red Antibody Labeling Reagent (Sartorius, 4723) at a molar ratio of 1:3 (test antibody: labeling Fab) in cell medium for 15 min at 37 °C. The antibody-FabFlour mixture was added to the cells to a final concentration of 2–8 µg/ml and the cell plate was directly transferred into an Incucyte S3 live cell analysis system (Sartorius) and images were obtained with a 10 × objective every hour for 48 h. Antibody internalization for each timepoint was calculated by normalizing the red fluorescent integrated intensity (after removal of isotype control IgG unspecific signal) to the phase confluency. All treatments were done in at least triplicates per concentration and condition, and expressed as mean normalized intensity ± S.D., from one representative experiment.

For cytotoxicity assays, anti-CXADR, anti-BSG, anti-CD47, anti-CD81, anti-CA9, or anti-FXYD6 antibodies at a fixed concentration (1.25 µg/ml for CXADR and 2.5 µg/ml for all other primary antibodies) were pre-complexed with anti-rabbit (αOIgG-CL-MMAF, Moradec LLC, AO-112AF) or anti-mouse IgG monomethyl auristatin F (αMFc-CL-MMAF, Moradec LLC, AM-102AF) ADC at a final concentration of 0.625–5 µg/ml in cell medium for 15 min at 37 °C. For control condition, no treatment, primary antibodies alone or untargeted primary antibodies (isotype control IgG; Invitrogen, Mouse: 31,903, Rabbit: 31,235) complexed with equivalent to the highest ADC concentration (5 µg/ml) were used. To assess cell cytotoxicity, Incucyte® Cytotox Red Dye (Essen Bioscience, 4632) was added to all samples at a final concentration of 250 nM. The ADC mixtures were added to the cells, keeping the hypoxic samples at 1% O_2_. The normoxic samples were transferred into the Incucyte S3 and images were obtained every 3 h for 96 h with a 10 × objective. Hypoxic samples were kept in the hypoxic incubator for 48, 72, and 96 h of treatment, transferred to the Incucyte, and images were directly acquired with the same settings as the normoxic samples.

Cytotoxic ADC effect was calculated by normalizing the red fluorescent area (cytotox area) to the phase confluency, and expressed as fold of cells only, mean ± S.D, from at least two independent experiments. For some experiments, cytotoxic effect was expressed as percentage of maximum cytotoxicity signal at 96 h. Statistical analyses were performed in GraphPad Prism using unpaired Student *t* test.

### Bioinformatics, graphics, and statistics

Visualization of differential gene expression analysis was generated with packages ggplot2 (v3.4.1), pheatmap (v1.0.12) clustered according to Ward’s minimum (ward.D2), and ggven (v0.1.9). Normoxic proteomic samples clustering was performed via Principal Component Analysis (PCA) with scaling using Singular value decomposition (SVD, within prcomp r function from stats r package, v4.2.2). Loading vectors (rotation values within prcomp output) were plotted for visualization of genes correlated with a principal component. For correlation between gene/protein abundances, cor function (stats r package) was applied to ranked abundances to perform Kendall’s Tau correlation of ranks. ComplexUpset (v1.3.3) r package was used to generate upset plots. All packages used are available at CRAN (https://cran.r-project.org/web/packages/) or Bioconductor repositories (https://bioconductor.org/). Data are expressed as the mean ± S.D. Statistical analyses were performed in R (4.3.1) within Rstudio environment (v2023.06.1–524, www.r-project.org and https://www.rstudio.com/) or GraphPad Prism (8.3.1), as indicated in the respective method. Wald test was used for significance inference of gene expression data; Kolmogorov–Smirnov statistics for GSEA results; one or two tailed unpaired Student t test for significance inference of flow cytometry data. Sample size (n) was determined by the number of experimental units analyzed (*i.e.*, number of genes, individual cell culture wells, technical replicates), as indicated in the respective figure legend. Results were normalized to corresponding controls. All values with *P* < 0.05 were considered statistically significant. Schematic figure was created with BioRender.com and figure composition was performed with Adobe Illustrator (27.9).

## Results

### The diverse surface-endocytome in GBM

**Fig. 1:**
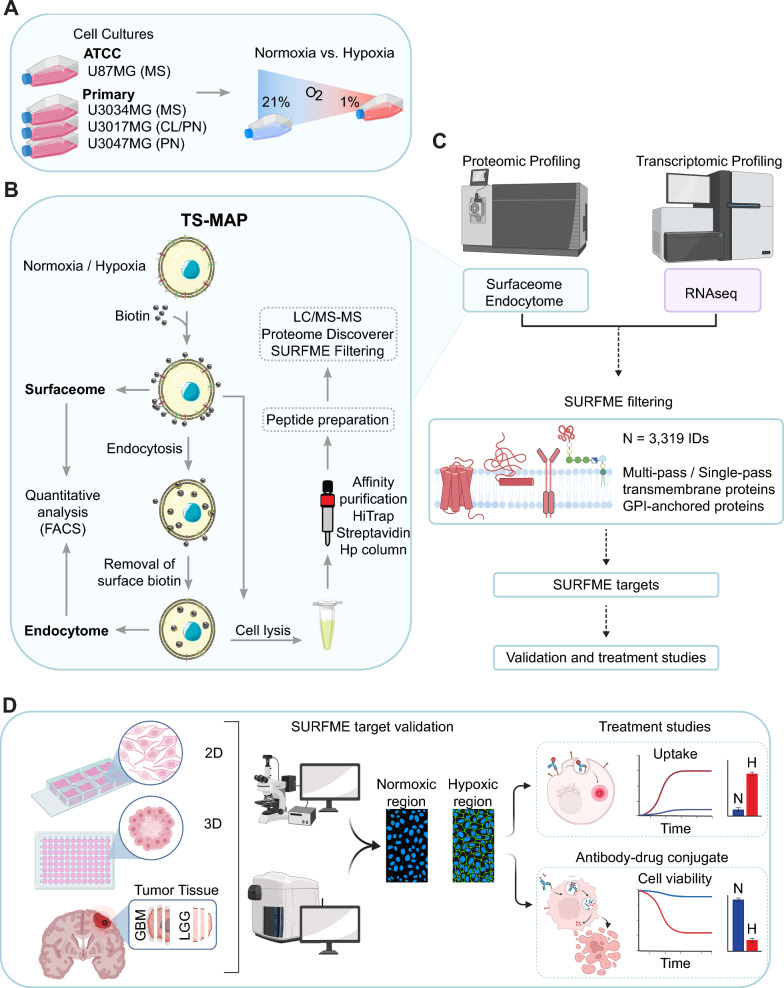
Comprehensive proteotranscriptomic profiling of the hypoxic surfaceome and endocytome in GBM. **A** Three primary GBM cell cultures representative of different molecular subtypes (*MS* mesenchymal, *CL* classical, and *PN* proneural) as well as the glioma cell line, U87MG, were cultured in normoxic or hypoxic conditions. **B** TS-MAP workflow for enrichment of the surfaceome and endocytome. **C** Quantitative LC–MS/MS of TS-MAP-enriched proteins and RNAseq from normoxic and hypoxic cultures. Data was filtered for *bona fide* plasma membrane proteins by the SURFME classifier. **D** Validation of target candidates by IF analyses of normoxic and hypoxic 2D cultures, spheroid (3D) hypoxic core, and comparative stainings of GBM and low-grade glioma (LGG; diffuse astrocytoma, WHO grade II) tumors, followed by selection for in vitro ADC treatment studies

**Fig. 2:**
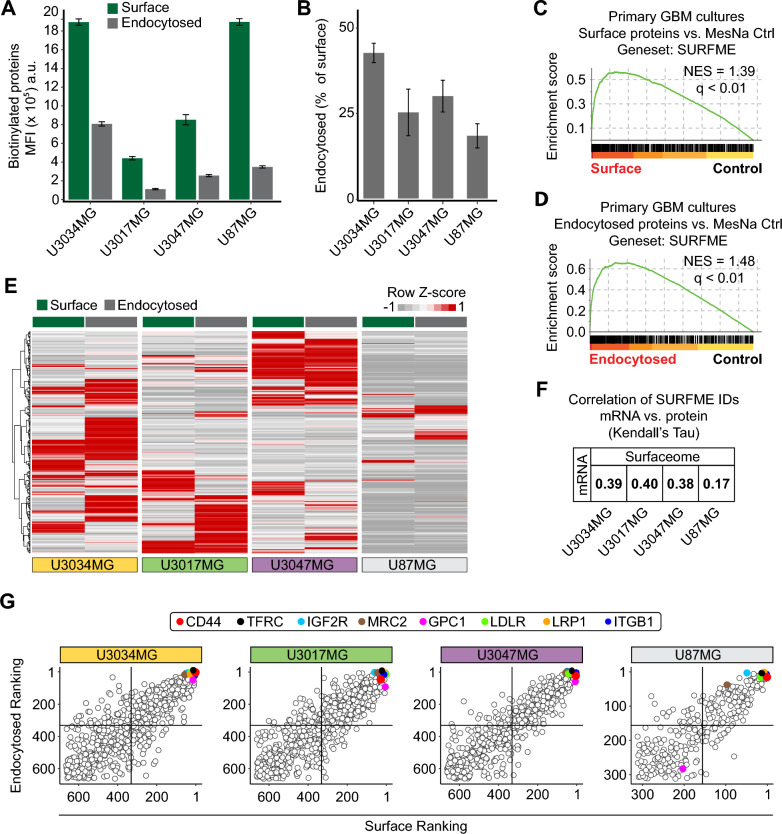
Identification of commonly abundant proteins among the diverse surface-endocytome in GBM. **A** Quantification of biotinylated surfaceome and endocytome in primary GBM cultures and U87MG cell line by FACS. **B** Fraction of endocytosed proteins expressed as the percentage of total surfaceome shown in (**A**). Data are presented as the mean ± S.D. from three independent experiments, each performed in triplicates. **C**, **D**, Analysis of LC–MS/MS profiling data confirms significant enrichment of SURFME protein identities by the TS-MAP approach in surface (**C**) and endocytome (**D**) samples when compared with control. **E** Heatmap of normalized TS-MAP data shows the heterogeneous abundance in surfaceome and endocytome identities between GBM cultures. **F** Kendall’s Tau correlation of SURFME identity ranks displays a weak association between transcriptomics and TS-MAP proteomics. **G** Ranking of SURFME proteins according to abundance (most abundant = first position) shows a strong association between surfaceome and endocytome in the respective GBM cell type. Top-right quadrants: proteins with high surface abundance and efficient endocytic capacity, highlighting some identities common across GBM cultures (see also, Additional file 4: Data File S3)

To profile the surface-endocytome in human GBM, we applied the TS-MAP approach on patient-derived, primary GBM IDH wildtype cultures (U3034MG, U3017MG, and U3047MG) [14, 18, 19] (Fig. 1). Primary GBM cultures were grown in serum-free medium to maintain stem cell-like capacity and subclonal diversity. To compare the subclonal diversity of primary cultures with differentiated glioma cells cultured in serum, we included the U87MG cell line derived from an IDH wildtype high-grade glioma with typical GBM mutations in the NF1, PTEN and TERT genes [15]. Cell expansion and hypoxic conditioning can trigger transcriptional drift and in primary cells [20]. However, primary GBM cultures and U87MG cells maintained their respective, original subtype throughout the experimental setup, as confirmed by RNASeq and GSEA (Additional file 1: Fig. S1A, and Additional file 2: Data File S1). TS-MAP profiling of primary and established glioma cells displayed efficient surface biotinylation, as shown by Western blot (Additional file 1: Fig. S1B-C) and, importantly, surface biotinylation was eradicated by MesNa treatment without compromising the integrity and biotinylation of endocytosed surface proteins (Additional file 1: Fig. S1D-E). By FACS analysis, we observed that the total surfaceome signal was almost 5- and 2.5-fold greater in U3034MG and U87MG compared to U3017MG and U3047MG, respectively (Fig. 2A, green bars). These results could be explained by differences in cell size in primary GBM cells (Additional file 1: Fig. S1F), although other factors such as differences in protein glycosylation also may contribute [21, 22]. However, surface protein abundance did not directly correlate with the global endocytic capacity (Fig. 2A, grey bars), which was highest in U3034MG (~ 40% of the surfaceome) and lowest in U87MG (~ 20%) when normalized to surface signal (Fig. 2B). To unveil the identities of surface and endocytosed proteins, we next proceeded with affinity chromatography enrichment of biotinylated proteins, followed by LC–MS/MS (Fig. 1B, C). As validation of the TS-MAP approach, GSEA of LC–MS/MS data revealed highly significant enrichment for the SURFME catalogue encompassing 3,319 cell-surface proteins (Additional file 3: Data File S2), in both surface and endocytosed protein fractions (Fig. 2C, D, and Additional file 1: Fig. S1G-H). SURFME includes single and multi-pass transmembrane proteins, as well as glycosyl-phosphatidyl-insositol (GPI)-anchored proteins from reviewed (Swiss-Prot) and manually annotated hits using the terms “cell membrane” and “extracellular domain” in UniProt (https://www.uniprot.org/) as well as from gene ontology (GO; http://geneontology.org/) terms “plasma membrane”, “cell surface”, and “external side of plasma membrane”. SURFME was then matched against the SURFY predictor (wlab.ethz.ch/surfaceome; [23]) which was considered to contain true hits. Importantly, SURFME contains several additional proteins (n = 433), not listed in the SURFY prediction algorithm, including the cell-surface marker CA9. We found that GBM cultures exhibit remarkably diverse surfaceome and endocytome identities with substantial cell-specific differences in protein abundances, as shown by hierarchical clustering of SURFME-filtered protein identities (Fig. 2E, and Additional file 4: Data File S3). This notion was substantiated by distinct surface-endocytome clustering in PCA analysis, *i.e.,* while U3017MG and U3047MG were closer together, U3034MG was clearly separated (Additional file 1: Fig. S1I and J). Moreover, the U87MG cell-line displayed a less complex profile than primary GBM cells (Fig. 2E), and PCA (Additional file 1: Fig. S1I and J) as well as SURFME category composition reflected a divergence between U87MG and primary cells (Additional file 1: Fig. S1K). Importantly, across all cell types, we consistently found weak correlations between RNAseq and LC–MS/MS, underscoring the limitations of transcriptomic analysis as a surrogate for quantifying membrane-associated surface proteins (Fig. 2F, and Additional files 2 and 4: Data Files S1 and S3).

We next ranked the surfaceome and endocytome proteins based on their relative abundances (Fig. 2G, and Additional file 4: Data File S3). From 664 SURFME protein identities quantified by TS-MAP, we uncovered numerous abundant surface proteins shared between primary GBM cells. These included cell-surface receptors involved in diverse functions, such as cell–matrix interactions (*e.g.,* ITGB1, GPC1, and CD44), membrane transport (*e.g.,* ATP1A1, LRP1, and LDLR), and immune responses (*e.g.,* HLA-A, SLC3A2, and ALCAM/CD166), many of which were also abundant in the endocytome (Fig. 2G, and Additional file 4: Data File S3). Additionally, we identified proteins that, while not top ranked at the cell surface, ranked high in the endocytosed fraction (*e.g.,* TFRC, IGF2R, and MRC2), suggesting their potential as proteins with elevated endocytic activity (Additional file 4: Data File S3). Together, our data highlight the diversity in global surfaceome composition and endocytic capacity, which is not mirrored by transcriptomic data in GBM. Interestingly, despite the inherent heterogeneity among patient-derived cell cultures, we identified a subset of proteins that consistently exhibited high abundance in both surfaceome and endocytome, serving as a valuable resource for future investigations.

### CD44 is a commonly abundant surface antigen with high endocytic capacity in GBM

**Fig. 3:**
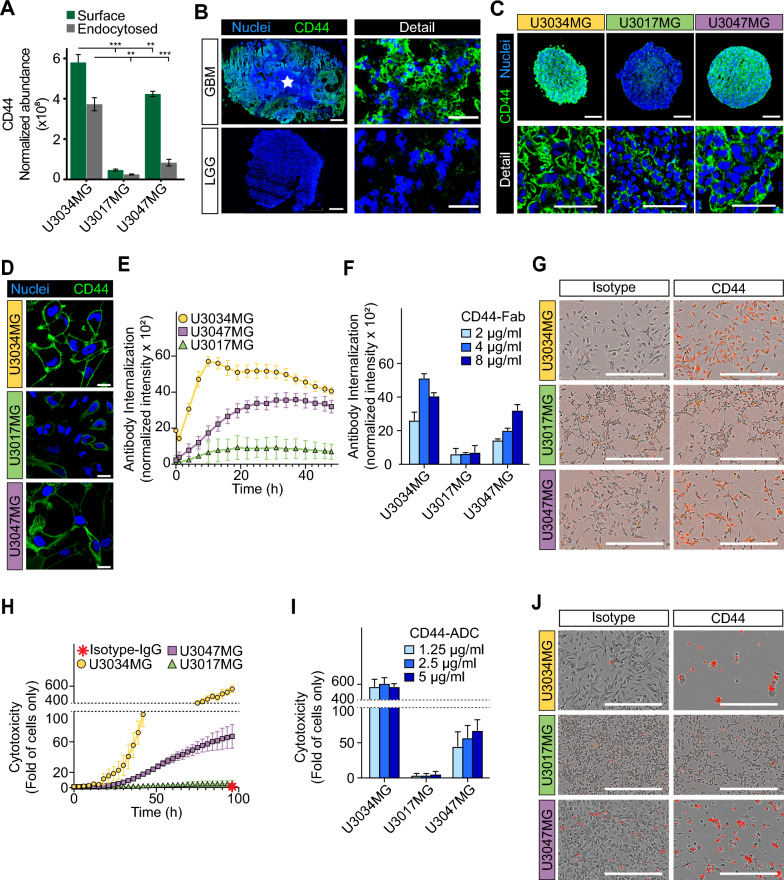
TS-MAP-based CD44 quantification directly correlates with the cytotoxic impact of ADC in GBM cells. **A** Comparative surface and endocytosed protein abundance between GBM cultures for the highly ranked CD44 TS-MAP candidate. *, **, *** *P* < 0.05, 0.01, and 0.001, respectively. **B**–**D** IF staining for CD44 in (**B**) GBM *vs.* LGG (white star: necrosis, scale bars, 1000 µm for scanned tissue sections, and 50 µm for confocal microscopy detail images) representative of at least three patients each, **C** spheroids (scale bars, 200 and 50 µm for scanned spheroid sections and confocal detail images, respectively), and **D** 2D cultures (scale bars, 20 µm), as indicated. Nuclei were stained with Hoechst (blue). **E** Internalization over time of Fabfluor pH Red-labelled CD44 antibody assessed by live-cell imaging of primary GBM cells, as indicated, and presented as red integrated intensity normalized to cell confluence. **F** Quantification of concentration-dependent anti-CD44 antibody internalization at 48 h. **G** Representative images of data (4 µg/ml) presented in (**F**) (scale bars, 450 µm). **H** Cytotoxicity over time analyzed by live-cell imaging of GBM cells treated with anti-CD44 ADC (5 µg/ml). Red star indicates no cytotoxicity by untargeted isotype control ADC. Cytotoxicity was calculated as red area normalized to confluency, and presented as fold of cells only ± S.D. from 2 independent experiments, each performed in triplicates. **I** Quantification of concentration-dependent cytotoxicity from (**H**) at 96 h of treatment. **J** Representative images of data shown in (**I**) (1.25 µg/ml; scale bars, 450 µm)

We next aimed to understand the utility of the TS-MAP approach for quantifying surface-endocytome proteins and explore its application for target identification in individual GBM cultures. By analyzing LC–MS/MS ranking data (Fig. 2G) for proteins associated with GBM aggressiveness, we identified CD44 as a proof-of-concept candidate with consistently high rankings (ranked 2/2 (surface/endocytosed; U3034MG), 5/23 (U3047MG), and 28/46 (U3017MG)). To corroborate these findings, we employed the TS-MAP protocol on an additional primary GBM culture (U3065MG, mesenchymal subtype), revealing CD44 as the most abundant SURFME protein, both on the cell-surface and in the endocytosed fraction (Additional file 4: Data File S3). CD44 (*alias* CSPG8) is a glycoprotein receptor that has been linked to glioma WHO grade, GBM cell stemness, invasion, poor prognosis and the mesenchymal subtype [24–26]. Consistent with global surface and endocytosed protein abundance and LC–MS/MS data, we observed significantly higher CD44 surface levels in U3034MG compared to U3017MG (~ 12-fold) and U3047MG (~ 1.3-fold) (Fig. 3A, green bars). Correspondingly, the fraction of endocytosed CD44 was ~ 15-fold and fourfold higher in U3034MG than in U3017MG and U3047, respectively (Fig. 3A, grey bars). Immunofluorescence analyses further suggested that CD44 is highly expressed in GBM while virtually absent in LGG (astrocytoma WHO grade II) (Fig. 3B). Importantly, we could verify that CD44 expression is highest in U3034MG, intermediate in U3047MG, and lower in U3017MG, by immunofluorescence analyses of spheroids (Fig. 3C) and 2D cultures (Fig. 3D).

We next explored how TS-MAP findings, that assesses constitutive endocytosis of CD44, corresponds with anti-CD44 antibody-triggered internalization. TS-MAP data was validated by quantitative live cell imaging, showing higher anti-CD44 antibody internalization kinetics and magnitude in U3034MG compared to U3047MG, while U3017MG exhibited minimal antibody internalization (Fig. 3E–G). We could show that these differences translated into dramatic variations in the sensitivity to anti-CD44 ADC treatment, with a ~ 300- and ~ tenfold higher cell killing effect in U3034MG compared to U3017MG and U3047MG, respectively (Fig. 3H–J). In U3034MG, maximum cell cytotoxicity was already obtained with the lowest ADC concentration (1.25 µg/ml) (Fig. 3I, light blue bar). Moreover, the kinetics of anti-CD44 ADC-induced cell killing were faster in U3034MG, reaching its maximum at ~ 60 h, while still increasing after 96 h of treatment in U3047MG (Additional file 1: Fig. S2A). U87MG cells, as expected from immunoflurescence analysis (Additional file 1: Fig. S2B-C), also showed efficient anti-CD44 antibody internalization (Additional file 1: Fig. S2D), and sensitivity to anti-CD44 ADC treatment (Additional file 1: Fig. S2E-F). Together, we provide proof-of-concept for the TS-MAP approach by revealing CD44 as an abundantly expressed target for efficient ADC delivery among the diverse surfaceome and functional endocytome in GBM.

### Hypoxic remodeling of the surface-endocytome in GBM

**Fig. 4:**
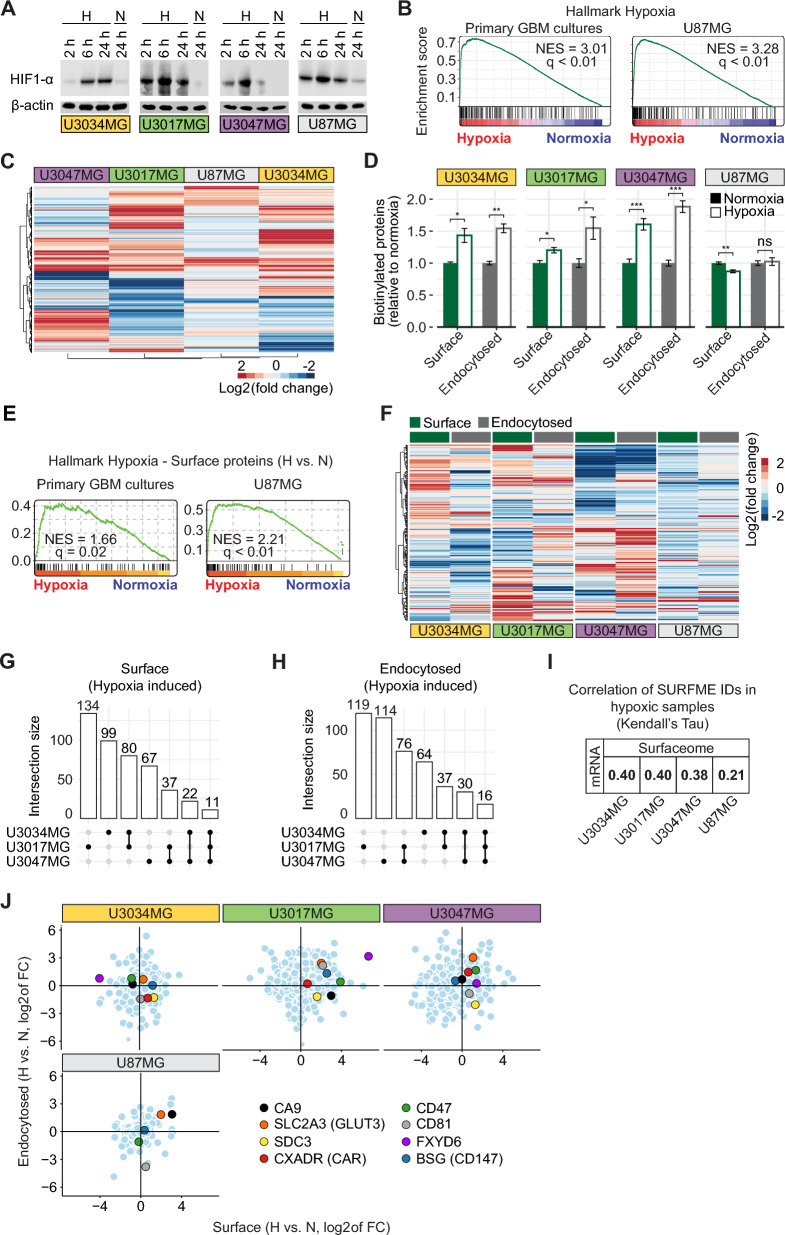
Hypoxic remodeling of the global surfaceome and endocytome in GBM cells. **A** Normoxic and hypoxic cell lysates at the indicated time-points were probed for HIF1-α by immunoblotting, with β-actin as loading control. **B** GSEA shows a significant overrepresentation of hallmark hypoxia-induced genes in mRNA of hypoxic *vs.* normoxic primary GBM (left) and U87MG (right) cells. **C** Heatmap of SURFME filtered mRNA data shows highly divergent hypoxic regulation between GBM cell types. **D** FACS quantification of global biotinylated surfaceome and endocytome in normoxic and hypoxic GBM cell cultures, as indicated. Data were normalized for normoxia (set at 1) and are expressed as the mean fold ± SD from three independent experiments, each performed in triplicates. *, **, *** *P* < 0.05, 0.01, and 0.001, respectively; ns, not significant. **E** GSEA on the surfaceome of hypoxic primary GBM (left) and U87MG (right) cells displays a significant enrichment of known hypoxia-responsive proteins when compared to the respective normoxic cells. **F** Hierarchical clustering of surface and endocytosed proteins identified in hypoxic compared to normoxic primary GBM and U87MG cells. **G**, **H** Upset plots show the distribution of common and unique hypoxia-induced surfaceome (**G**) and endocytome (**H**) proteins in GBM cells. **I** Kendall’s Tau correlation of hypoxia-induced IDs indicates weak associations between SURFME-filtered mRNA and protein abundance. **J** SURFME proteins identified in the surfaceome as well as the endocytome of GBM cells, ranked according to log_2_ of the fold change of hypoxia *vs.* normoxia and divided into quadrants. Top-right, proteins with higher surface abundance and more efficient endocytic capacity in hypoxic conditions

Hypoxia strongly contributes to GBM aggressiveness and resistance to conventional treatment modalities, which motivates efforts to map the hypoxia-induced surface-endocytome with the potential of tailoring therapies directed at this specific niche. We found a robust hypoxic response across all primary GBM cultures and U87MG cells, substantiated by HIF1-α protein stabilization (Fig. 4A) and GSEA (Fig. 4B). Hierarchical clustering based on differentially expressed genes (DEGs) displayed a heterogeneous global response to hypoxia among GBM cell types (Additional file 1: Fig. S3A-C). Notably, the SURFME-filtered transcriptome was profoundly remodelled by hypoxia (Fig. 4C), with in total 909, 829, and 800 up or down-regulated genes in U3017MG, U3034MG, and U3047MG, respectively. U87MG displayed relatively limited regulation by hypoxia (in total 305 up or down-regulated genes), probably due to subclonal restrictions. Nevertheless, we could identify several SURFME genes universally induced by hypoxia in all cell types, including U87MG. Most of these genes encode for proteins involved in pH-regulation and glucose transport (Additional file 1: Fig. S3D).

We next explored the dynamic influence of hypoxia over time on the global surfaceome and endocytome. FACS quantification indicated that short-term hypoxia (2 and 6 h) had a negative effect, especially on endocytosis (Additional file 1: Fig. S4A). Notably, however, at prolonged hypoxia (24 h), we consistently found a significant increase in global surfaceome and endocytome abundance, a phenomenon not observed in U87MG cells (Fig. 4D). With the aim to identify hypoxia-induced tumor antigens, and assuming that the perinecrotic niche of GBM tumors is exposed to prolonged hypoxia, we choose the 24 h time-point for further studies. We could show that hypoxic conditioning for 24 h is significantly reflected in the surfaceome (Fig. 4E), impacting, as well, the categories of SURFME proteins expressed in the surface-endocytome (*cf.* Additional file 1: Fig. S4B and Additional file 1: Fig. S1K). In accordance with global FACS quantification (Fig. 4D) and heat map visualization (Fig. 4F), we showed that U87MG cells are less responsive with in total *n* = 41/46 (surface/endocytosed) hypoxia induced SURFME proteins, as compared with U3017MG (*n* = 262/248), U3034MG (*n* = 212/147), and U3047MG (*n* = 137/236) (Additional file 1: Fig. S4C). The diverse hypoxic response was underscored by the limited number of shared identities between primary cultures, with *n* = 11 in the surfaceome and *n* = 16 in the endocytome (Fig. 4G and H). When comparing the degree of hypoxic induction between mRNA and proteomics, we again found that transcriptomic analysis poorly predicted hypoxic regulation at the protein level (Fig. 4I). To better visualize the diverse hypoxic response, we next ranked surfaceome and endocytome identities based on their fold hypoxic induction in the respective cell type (Fig. 4J, and Additional file 4: Data File S3). We could reveal commonly hypoxia-induced proteins in GBM, such as CXADR and SDC3 in the surfaceome, and CD47 and FXYD6 in the endocytome, while SLC2A3 (GLUT3) was up in both the surfaceome and endocytome.

### Validation of hypoxia-induced tumor antigens for ADC toxicity studies

We next set out to validate hypoxia-induced cell-surface proteins identified by TS-MAP. We could show that SLC2A3 protein expression is consistently induced in hypoxic GBM cultures, both in 2D and within the hypoxic core of spheroids (Additional file 1: Fig. S5A and B). Notably, SLC2A3 expression was highly induced in the perinecrotic/hypoxic area of GBM tumor sections, while exhibiting relatively sparse expression in LGG tumors that inherently lack perinecrotic regions (Additional file 1: Fig. S5C). SLC2A3 consists of 12 transmembrane segments with few extracellular epitopes and is predominantly expressed in the brain, which makes it a challenging target even for local, intratumoral immunotherapies. CA9, a single-pass transmembrane receptor, is currently explored as a promising target for small molecule inhibitors and antibody-based cancer treatments [27]. We found a marked increase in CA9 protein expression under hypoxic conditions in both 2D and 3D U87MG cell cultures (Additional file 1: Fig. S5D and E), as well as robust expression in GBM while virtually absent in LGG tumors (Additional file 1: Fig. S5F). This differential expression pattern translated into increased susceptibility of hypoxic cells to anti-CA9 ADC treatment, while normoxic cells were resistant across all tested concentrations (Additional file 1: Fig. S5G-H).

**Fig. 5:**
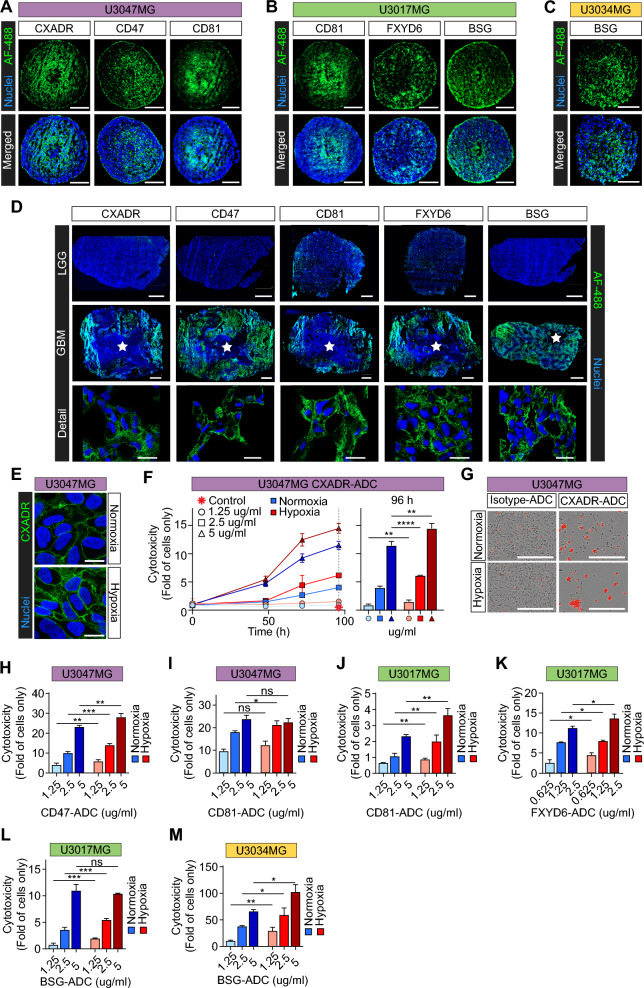
Validation of hypoxia-induced tumor antigen targets for ADC toxicity studies. **A**–**C** Representative IF stainings for selected, hypoxia-induced targets, as indicated, in GBM spheroids derived from (**A**) U3047MG, **B** U3017MG, and **C** U3034MG primary cells (scale bars, 200 μm). **D** Validation of hypoxia-induced targets in GBM and LGG tumors, as indicated (white star: necrosis, scale bars, 1000 and 20 μm for scanned sections and confocal microscopy detail images, respectively). Data shown is representative of at least three patients each. **E** Increased CXADR protein expression in hypoxic *vs.* normoxic U3047MG 2D cultures, assessed by confocal microscopy (scale bars, 20 μm). **F** Left: Cytotoxicity over time by different concentrations of anti-CXADR ADC (isotype control ADC, red star). Cytotoxicity was calculated as red area normalized to confluency, and presented as fold of cells only ± S.D. from 2 independent experiments, each performed in triplicates. Right: Quantification of cytotoxicity at 96 h of treatment. **G** Representative images from data shown in (**F**) (5 µg/ml, at 96 h; scale bars, 450 µm). **H**–**M** Quantification of cytotoxicity of anti-ADC treatments for 96 h at hypoxia and normoxia, directed against (**H**) CD47 and (**I**) CD81 in U3047MG cells; (**J**) CD81, **K** FXYD6, and **L** BSG in U3017MG cells; and **M** BSG in U3034MG cells. *, **, *** *P* < 0.05, 0.01, and 0.001, respectively; ns, not significant. IF images are representative of at least 2 independent experiments

To explore potential new cell-surface antigens induced by hypoxia, we focused on proteins with divergent normalized relative abundances between hypoxia and normoxia according to TS-MAP data. We decided to select five candidates for validation experiments: CXADR, known as a receptor for coxsackievirus and adenovirus that has shown potential to promote tumorigenesis and as a target for oncolytic viral therapy [28–30]; CD81, known as a receptor of HCV that has been implicated in tumor progression and as a potential immunotherapeutic target in lymphoma and breast cancer [31–34]; CD47, suggested as a targetable immune checkpoint in GBM and other malignancies, and was shown to be hypoxia/HIF1α-regulated in breast cancer [35–37]; BSG, shown to be overexpressed and hypoxia-induced in GBM [38], and was suggested as an ADC target in hepatocellular carcinoma [39]; and FXYD6, which is a relatively unexplored regulator of the Na + /K + -ATPase, and was suggested as therapeutic target in cancer [40]. In all cases, we could successfully validate TS-MAP data by showing increased surface expression in hypoxia, in both GBM spheroid cores and 2D cultures (Fig. 5A–C, and Additional file 1: Fig. S6A). Moreover, we observed abundant expression of selected proteins in the perinecrotic/hypoxic regions of GBM, while their expression was low (CD81 and FXYD6) or absent (CXADR, CD47, and BSG) in LGG tumors (Fig. 5D). Moreover, in all cases, we found no detectable expression in normal brain (Additional file 1: Fig. S6B). Notably, selected proteins preferentially located to the cell-surface in GBM tumors (Fig. 5D, detail). We next focused on CXADR, first described as a receptor for oncolytic adenoviruses by Douglas et al*.* [41], showing robust induction in both spheroid core area (Fig. 5A, left panel), and hypoxic *vs.* normoxic U3047MG 2D cultures (Fig. 5E). Remarkably, we found efficient, dose and time-dependent anti-CXADR ADC cytotoxicty and, across all concentrations tested, the effect was significantly greater in hypoxia as compared with normoxia (Fig. 5F). As a control, treatment with an isotype IgG ADC under similar conditions displayed no cytotoxicity in either hypoxia or normoxia (Fig. 5F, red star, and Fig. 5G). In U3047MG, we similarly found significantly increased sensitivity to anti-CD47 ADC in hypoxia *vs.* normoxia over the entire range of concentrations (Fig. 5H, and Additional file 1: Fig. S6C). While specifically lower concentrations of anti-CD81 ADC were more efficient in hypoxic U3047MG cells (Fig. 5I, and Additional file 1: Fig. S6C), anti-CD81 ADC consistently showed greater toxicity (up to ~ twofold) in hypoxic *vs.* normoxic U3017MG at all concentrations (Fig. 5J, and Additional file 1: Fig. S6D). This observation aligns well with TS-MAP ranking data, showing a relatively higher hypoxic induction of CD81 in U3017MG (⁓4.5-fold up in surfaceome) as compared with U3047MG (⁓1.6-fold up) (Additional file 4: Data File S3). Further, CD81 was ⁓4.5-fold up in the hypoxic *vs*. normoxic U3017MG endocytome, whereas in U3047MG it was rather downregulated (Fig. 4J, and Additional file 4: Data File S3). We also found a comparable hypoxic sensitization to anti-FXYD6 ADC (Fig. 5K, and Additional file 1: Fig. S6D) and anti-BSG ADC (Fig. 5L, and Additional file 1: Fig. S6D) treatments in U3017MG. We could corroborate preferential targeting of BSG in hypoxic U3034MG, demonstrating up to ~ threefold higher sensitivity to anti-BSG ADC as compared with normoxic conditions (Fig. 5M, and Additional file 1: Fig. S6E). Together, we identify several hypoxia-induced ADC target proteins, CXADR, CD47, CD81, BSG, and FXYD6, and provide proof-of-concept evidence that TS-MAP can address the distinct hypoxic adaptations observed across GBM cultures from individual tumors.

## Discussion

The development of immunotherapies for cancer, specifically employing CAR-T cells and ADCs, relies on an improved understanding of the cancer cell surfaceome. Here, we have performed comprehensive proteotranscriptomic mapping to unveil the diverse surface-endocytome and its remodeling by hypoxia in GBM. We identify several surface proteins exhibiting high endocytic activity that are consistently abundant, offering a valuable resource for target discovery in GBM. We reveal how hypoxic stress profoundly reshapes the GBM surface-endocytome, and validate several potential ADC target candidates; CXADR, CD47, CD81, BSG, and FXYD6. On a more fundamental level, our results may shed light on how hypoxic surfaceome remodelling can contribute to GBM progression with implications for cell–cell and cell–matrix interactions.

GBM exhibits a complex microenvironment that cannot be fully replicated in vitro. Our approach was focused on delineating the isolated effect of hypoxia in GBM. For our hypoxia studies, we choose 1% O_2_, which is a widely standardized oxygen level that recapitulates the hypoxic situation in tumors and is known to stabilize the hypoxia master regulator HIF1α, as verified in the present study. For normoxic controls, routine culture conditions (5% CO_2_: 95% air) were applied, which corresponds to ⁓20% O_2_. The pO_2_ of human blood normally ranges 10–13% O_2_ [42], and in the brain cortex it may be as low as 5–6% O_2_ [43]. Importantly, however, we could validate TS-MAP findings by increased expression of identified proteins in the hypoxic spheroid core as well as in GBM as compared with LGG tumors that are considerably less hypoxic. Moreover, for all candidate target proteins, we found no detectable expression in normal brain.

Increased expression of surface receptors and endocytic activity in hypoxia primarily serves to fulfill an increased metabolic demand [44]. Consistent with this notion, we demonstrate that primary cultures grown in serum-free conditions upregulate their surface-endocytome during hypoxic stress, and SLC2A3/GLUT3 was commonly induced in all cell types. The induction of GLUT transporters (SLC2A1/GLUT1 and SLC2A3) to support aerobic glycolysis (the Warburg effect) is well established [4], a phenomenon that is reinforced by HIF-1/2α transcriptional control in the hypoxic niche of malignant tumors. HIFs, *i.e*., key sensors of intracellular oxygen availability [7], can also induce lipid uptake through increased expression of lipid receptors (CD36 and FABP4) [45, 46]. In glioma, however, hypoxia was shown to induce membrane raft-dependent endocytosis of lipid particles via HIF-1/2α-independent mechanisms [7, 47]. Thus, hypoxic regulation of endocytic uptake may occur through either HIF-1/2α-mediated or alternative mechanisms. Among identified proteins, only CD47 and BSG have previously been associated with hypoxic induction and HIF regulation [37, 38]. Notably, hypoxia over 24 h, as applied in the present study, may also entail indirect, paracrine effects imposed by secreted growth factors and cytokines. The extent to which HIFs and paracrine mechanisms dictate hypoxic modulation of the surface-endocytome in cancer remains an interesting avenue for future investigations.

The emergence of ADCs has brought the concept of exploiting the tumor surfaceome for cytotoxic cargo delivery into the spotlight. Currently, six ADCs are FDA-approved for the treatment of solid tumors, and numerous trials are ongoing. So far, CAR-T cell therapy, that was not explored in the present study, has been approved for haematological malignancies, while in solid tumors promising effects are confined to experimental models and a limited number of patients [48]. The variable expression of target proteins poses a particular challenge for ADC therapies, calling for more rational multi-targeting strategies with *e.g.,* bispecific antibodies that still are under development. However, ADCs can be designed to carry a lipid-soluble payload and a cleavable antibody linker, allowing bystander effects in tumor cells devoid in target expression. Whereas CAR-T cell treatments prefer surface resident targets, ADCs rely on efficient endocytosis. Using the TS-MAP approach, we provide insights into this key aspect by presenting a comprehensive ranking of surface residence *vs.* endosomal transition, encompassing more than 600 SURFME identities from hypoxic and normoxic conditions. While the endocytic capacity of cell-surface proteins may help to predict their suitability as ADC targets, it should be acknowledged that antibody-induced clustering and internalization may differ from constitutive endocytosis, and ADC induced endocytosis needs to be carefully evaluated for every individual target-ADC pair.

Within the vast surfaceome heterogeneity, we identify CD44 as a common and abundantly expressed surface antigen with high endocytic capacity to deliver ADC drugs that should stimulate intensified efforts in the development of CD44-targeteted ADCs in GBM treatment. Notably, CD44 exists as several splice variants that may be more or less tumor specific, and future analyses should clarify which variants are dominant in GBM cells. Further, CD44 is expressed by non-tumor cells, including hematopoietic progenitors, which may pose challenges in clinical translation. However, the fact that presently approved ADCs indeed target proteins (including HER2, TF/F3, and TROP2) known to be expressed in numerous normal tissues, we conclude that in vivo studies will be required to reveal potential unspecific toxicities and adverse events. Such studies should include a theranostics approach, *e,g,* using radioisotope-conjugated antibodies for PET-MRI evaluation of target protein expression in tumor *vs.* normal tissues.

By integrating TS-MAP proteomic profiling and transcriptomic analysis, we highlight the discordance between mRNA and actual membrane protein levels. Plasma membrane proteins undergo various post-translational modifications, including glycosylation that significantly affects their localization and turnover rate [21, 22]. Also, factors like ribosome availability, alternative splicing and microRNA-mediated inhibition influence translation efficiency. On the technical side, RNA sequencing and proteomic approaches have varying sensitivity and dynamic range, *i.e.* mass spectrometry could be biased against less abundant proteins, contributing to the discrepancies. For example, EGFRvIII, PDGFRα, and MET, which are implicated in gliomagenesis and as GBM targets, may be poorly detectable by proteomics.

An obvious obstacle to efficient antibody and CAR-T cell delivery to brain tumors is posed by the BBB [49]. Notably, trastuzumab deruxtecan showed clinically relevant responses against brain metastasis in a phase II study with breast cancer patients [50, 51]. Depatuxizumab mafodotin, an ADC targeting EGFR and EGFRvIII, showed objective radiological responses, but did not provide significant overall survival benefit in GBM patients [51]. As recently suggested, [52] optimizing ADC homogeneity and drug-to-antibody-ratio (DAR), may allow efficient ADC delivery into GBM tumors. Moreover, microbubble-assisted low-intensity pulsed focused ultrasound (LIFU) has shown promise in temporarily opening the BBB and facilitating the delivery of therapeutic agents [49], *e.g.* a recent GBM trial reported an increased BBB passage of cytostatic drugs [53]. In support of enhanced BBB penetration of ADCs, LIFU facilitated intracranial delivery of trastuzumab emtansine in experimental brain metastasis [54]. Clearly, future efforts should be focused on evaluating treatment effects in relevant in vivo models that explore *e.g.,* stereotactic ADC injections directly into the tumor, or combinations with LIFU, radiotherapy and other strategies to overcome the BBB.

## Conclusions

This study uncovers the heterogeneity of the surface-endocytome and the impact of hypoxia on its remodeling in GBM. The identification of abundantly expressed antigens and the exploration of hypoxia-driven alterations may open new avenues for immunotherapies and targeted cytotoxic therapies in GBM. Moreover, our findings underscore the value of targeted proteomics in dissecting the complexity of the surface-endocytome and identifying potential therapeutic targets. It will be interesting to expand on these studies in a larger cohort, to better understand the underlying mechanisms that dictate the surfaceome heterogeneity and its dependence on GBM transcriptional subtypes and driver mutations.

### Supplementary Information

**Additional file 1**: Supplementary Figures.

**Additional file 2**: RNA seq data.

**Additional file 3**: SURFME catalogue.

**Additional file 4**: Proteomics data.

## Data Availability

Transcriptomic data generated in this manuscript are deposited at the Gene Expression Omnibus (GEO) database and can be accessed at https://www.ncbi.nlm.nih.gov/geo (accession codes: GSE245800 and GSE245762). The mass spectrometry proteomics data have been deposited to the ProteomeXchange Consortium via the PRIDE [55] partner repository with the dataset identifier PXD048880. Further supplementary materials are provided in Data Files  S1-4.

## Abbreviations

ADC: Antibody–drug-conjugates
GBM: Glioblastoma
BTB: Blood-tumor-barrier
TS-MAP: Tumor surfaceome mapping
LGG: Low grade glioma
HGCC: Human glioma cell culture
DMEM: Dulbeco’s modified Eagle medium
MesNa: Sodium-2-mercaptoethanesulfonate
IAA: Iodacetamide
TCA: Trichloroacetic acid
RT: Room temperature
AMBIC: Ammonium-bicarbonate
TFA: Trifluoroacetic acid
ACN: Acetonitrile
UHPLC: Ultrahigh pressure liquid chromatography
DDA: Data-dependent acquisition
AGC: Automatic gain control
HCD: High-energy collision dissociation
NCE: Normalized collision energy
O/N: Overnight
BEA: Bioinformatics and expression analysis
GSEA: Geneset enrichment analysis
RIPA: Radioimmunoprecipitation assay
PVD: Polyvinylidene difloride
TTBS: Tris-buffered Saline with Tween
HRP: Horseradish peroxidase
ECL: Enhanced chemiluminescence
BSA: Bovine serum albumin
FACS: Fluorescence-activated cell sorting
PFA: Paraformaldehyde
AF: Alexa fluor
MFI: Mean fluorescence intensity
OCT: Optimal cutting temperature
αMFc-CL-MMAF: Anti-mouse IgG monomethyl auristatin F
PCA: Principal component analysis
SVD: Singular value decomposition
LIFU: Low-intensity pulsed focused ultrasound
DEG: Differentially expressed genes
STR: Short-tandem repeat
PLO: Poly-L-ornithine
αOIgG-CL-MMAF: Anti-rabbit IgG monomethyl auristatin F
FDA: Food and Drug Administration
DAR: Drug-to-antibody ratio
BBB: Blood–brain barrier
