# Supplementary material for: Decoding of the surfaceome and endocytome in primary glioblastoma cells identifies potential target antigens in the hypoxic tumor niche
Source: Acta Neuropathol Commun. 2024 Feb 27;12:35. doi: 10.1186/s40478-024-01740-z (PMC10898066; doi:10.1186/s40478-024-01740-z)
Supplement: Supplementary file 1 — Additional file 1: Supplementary Figures. [file 40478_2024_1740_MOESM1_ESM.docx]

**Decoding of the surfaceome and endocytome in primary glioblastoma cells identifies potential target antigens in the hypoxic tumor niche**

Kelin Gonçalves de Oliveira, Anna Bång Rudenstam, Sarah Beyer, Axel Boukredine, Hugo Talbot, Valeria Governa, Maria C. Johansson, Ann-Sofie Månsson, Karin Forsberg Nilsson, Johan Bengzon, Johan Malmström, Charlotte Welinder, Mattias Belting

**Supplementary Figures and Legends**

**
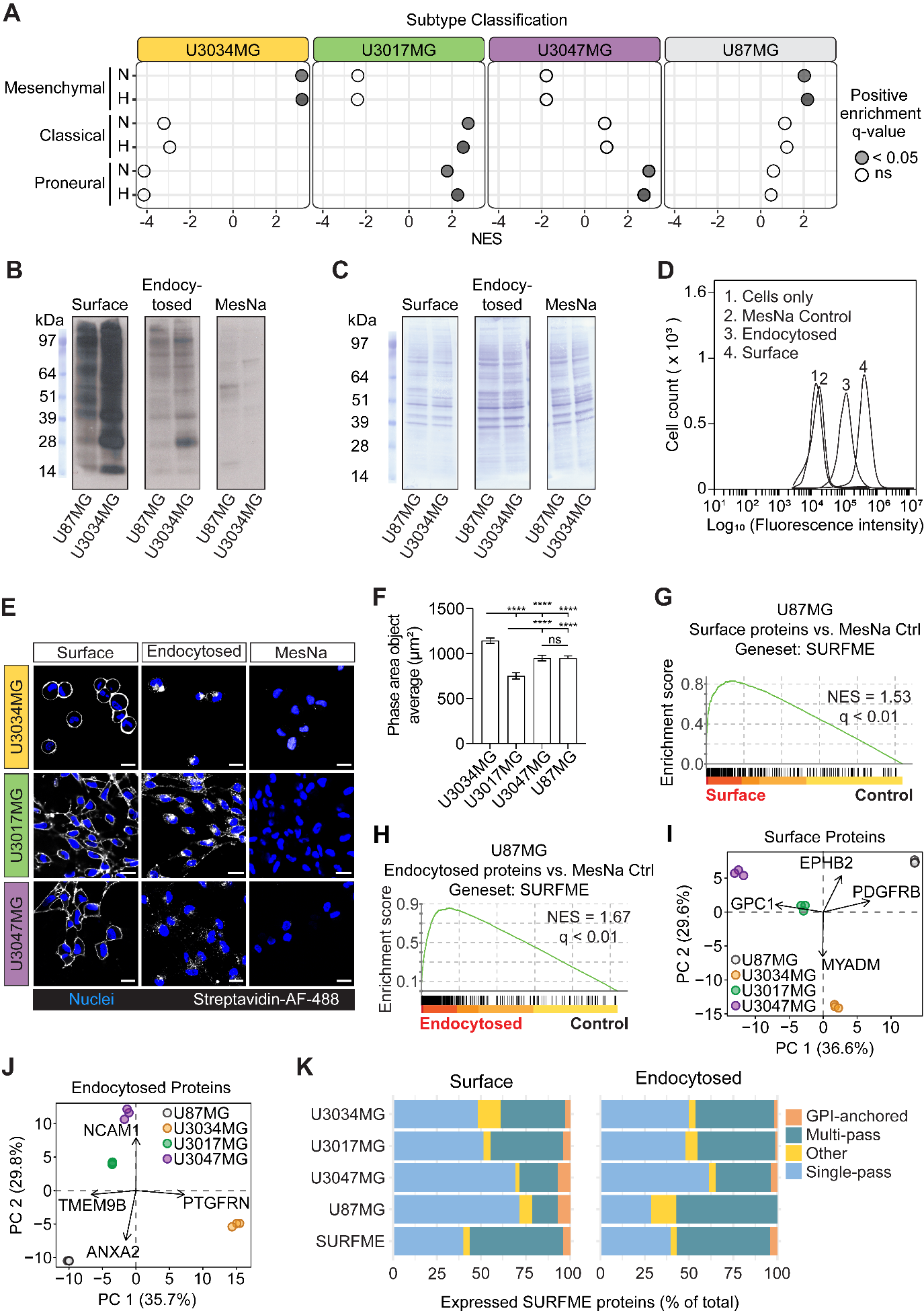
**

**Fig. S1. A**) GBM transcriptional subtype enrichment plots (Verhaak, *et al.,* 2010) for normoxic (N) and hypoxic (H) cells show no apparent influence of hypoxia on subtype classification in primary GBM cultures or U87MG cells. **B**) Western blotting shows efficient biotinylation of surface and endocytosed proteins in primary (U3034MG) and U87MG GBM cells, and efficient removal of surface labeling by MesNa. **C**) Coomassie blue stained membrane as equal loading control for western blotting data presented in (**B**). **D**) Representative FACS plots for: (1) non-biotinylated cells, (2) residual surface biotin signal after MesNa treatment, (3) endocytosed surface proteins, and (4) total surface biotin signal. **E**) Confocal microscopy images representative of experimental groups of the TS-MAP protocol (Surface, Endocytosis, MesNa) in all three primary GBM cells (scale bar, 20 μm) from 2 independent experiments, each performed in triplicates. **F**) Cell-by-cell analysis displaying cell size presented as average phase area object ± S.D. from 8-12 wells/cell-line. **** P < 0.001, ns = not significant. **G**) and **H**) Analysis of TS-MAP profiling data, displaying significant enrichment of SURFME protein identities in U87MG cells in (**G**) surfaceome and (**H**) endocytome samples when compared with control samples. **I**) and **J**) PCA show sample distribution along the first two principal components based on TS-MAP data of surface proteins (**I**) and endocytosed proteins (**J**) from normoxic cells. Arrows and gene symbols indicate PC direction and the identities of the most important proteins contributing to the given variance, respectively. **K**) Categories of SURFME subgroups represented in the surfaceome and endocytome of normoxic GBM cells.


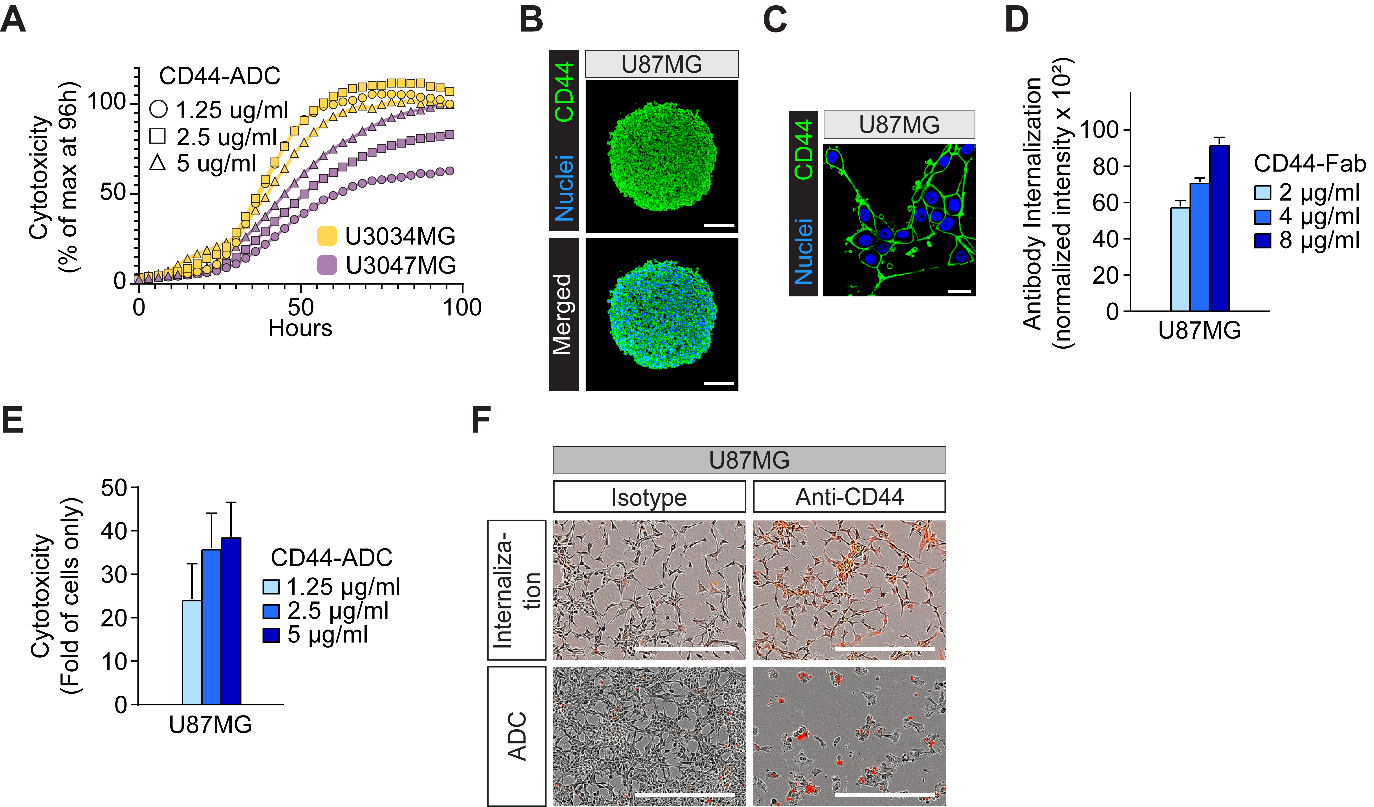


**Fig. S2.** **A**) Dose and time-dependent cytotoxicity of anti-CD44 ADC reveals increased cell killing kinetics in U3034MG, reaching its maximum at ~ 60 h, while still increasing after 96 h of treatment in U3047MG. Data are expressed as percentage of maximum cytotoxicity signal at 96 h. **B** and **C**) IF staining for CD44 in U87MG (**B**) spheroid (scale bars, 200 µm) and (**C**) 2D culture (scale bar, 20 µm). **D**) Internalization of Fabfluor pH Red-labelled CD44 antibody by U87MG cells at 48 h, assessed by live cell imaging, and presented as red intensity normalized to cell confluency. **E**) Quantification of concentration dependent anti-CD44 ADC cytotoxicity at 96 h of treatment. Cytotoxicity was calculated as red area normalized to confluency and presented as fold of cells only ± S.D. from 2 independent experiments, each performed in triplicates. **F**) Representative live-cell images of experiments described in (**D**) and (**E**). Upper row: CD44-Fab antibody internalization (4 µg/ml). Bottom row: Anti-CD44 or isotype control ADC treatment (1.25 µg/ml) (scale bars, 450 µm).


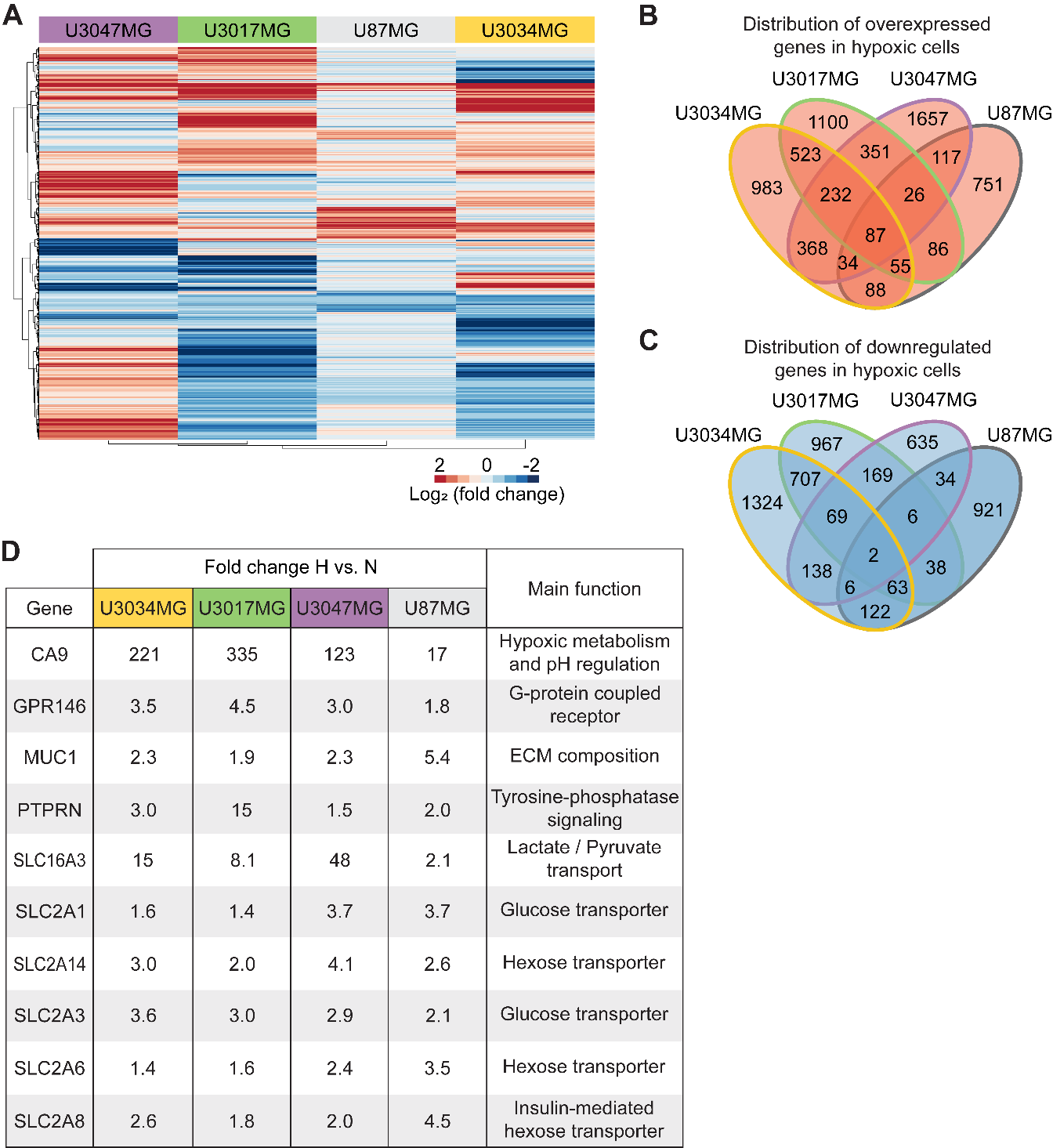


**Fig. S3. A**) Hierarchical clustering of RNASeq displays divergent hypoxic regulation in primary GBM and U87MG cells. **B**) and **C**) Venn diagrams show distribution of commonly and uniquely upregulated (**B**) and downregulated (**C**) genes in different GBM cell types, as indicated. **D**) Selection of SURFME mRNAs commonly upregulated in hypoxic (H) *vs*. normoxic (N) GBM cells.


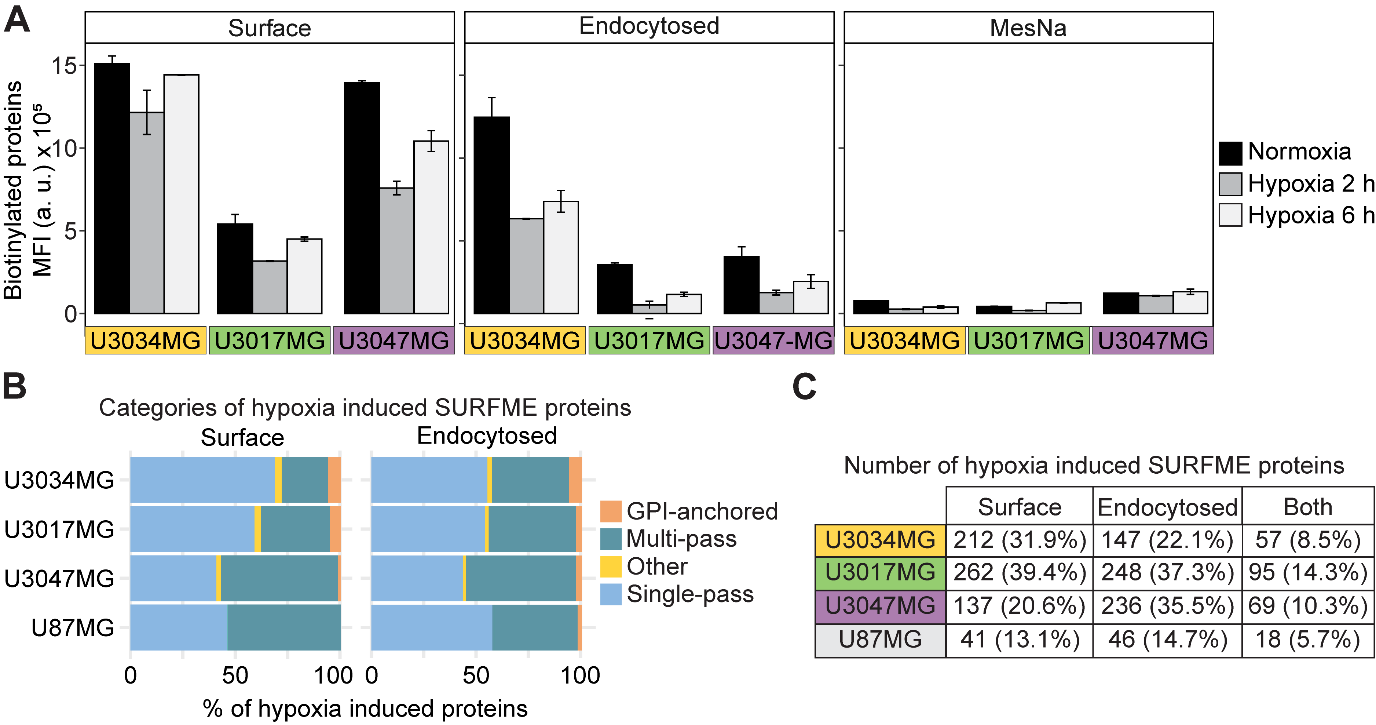


**Fig. S4.** **A**) FACS quantification of global biotinylation (Surface, Endocytosis, and MesNa control) in primary GBM cells conditioned in normoxia, and 2 or 6 h of hypoxia. Data were normalized to controls and presented as MFI ± S.D. from triplicate experiments. **B**) Categories of SURFME subgroups represented in the hypoxia-induced surfaceome and endocytome in the respective GBM cell type. **C**) Number of SURFME proteins upregulated in hypoxic GBM cells compared to normoxic controls, in the surfaceome, endocytome, or both. Inside brackets - the fraction of SURFME proteins induced by hypoxia, expressed as a percentage of the total number of SURFME proteins identified in each cell-line.


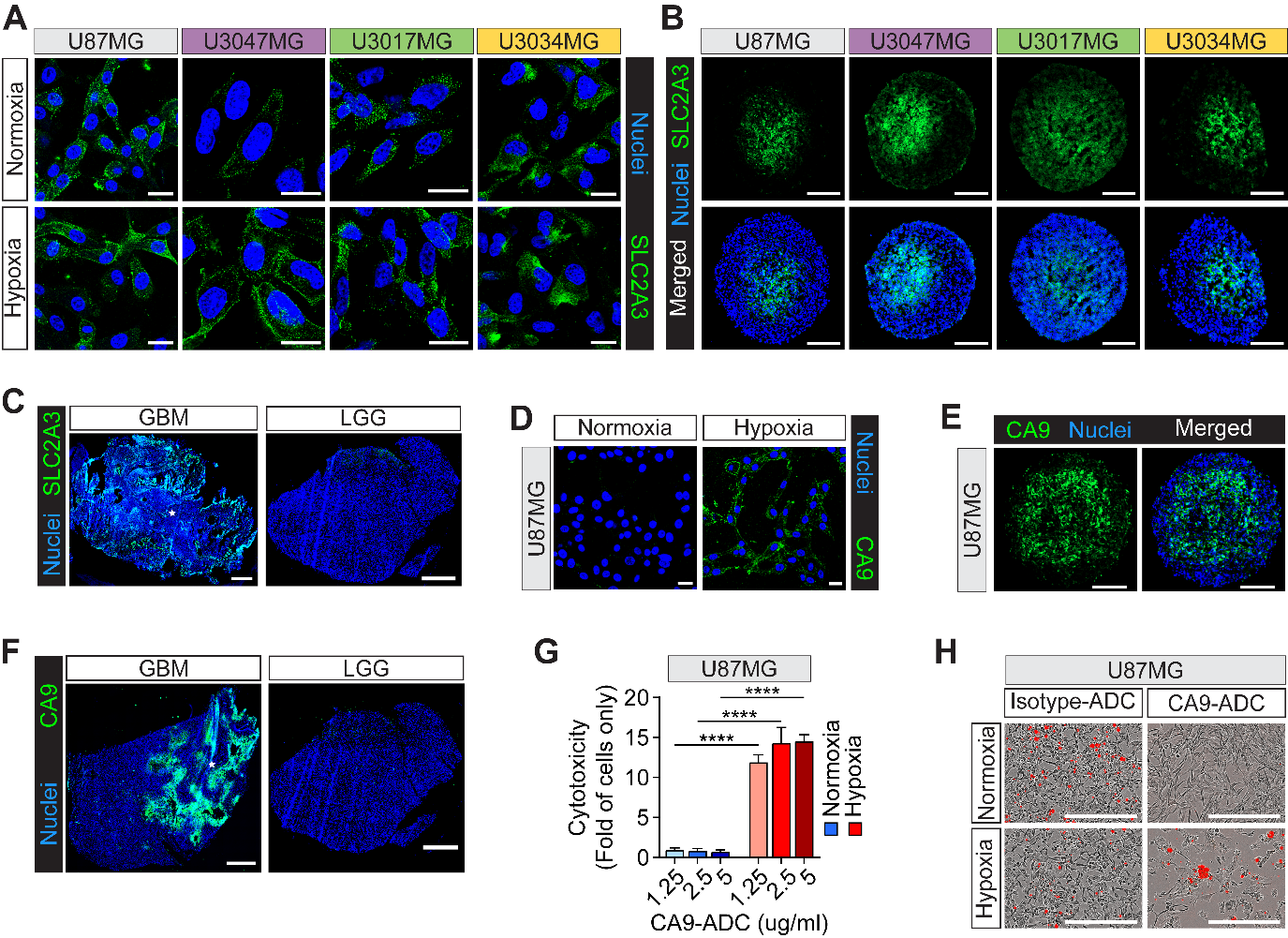


**Fig. S5.** **A**-**C**) IF stainings for SLC2A3 in (**A**) hypoxic and normoxic GBM 2D cultures, assessed by confocal microscopy (scale bars, 20 µm), (**B**) GBM spheroids and (**C**) GBM and LGG tumors, assessed by slide scanner (scale bars, 200 and 1000 µm, respectively). **D**-**F**) Similar experiments as in (**A-C**) with IF staining for CA9 in (**D**) hypoxic and normoxic U87MG 2D cultures, (**E**) spheroids, and (**F**) GBM and LGG tumors. **G**) Cytotoxicity at 96 h of anti-CA9 ADC treatment at the indicated concentrations, in normoxic and hypoxic U87MG cells. Cytotoxicity was calculated as red area normalized to confluency and presented as fold of cells only ± S.D. from triplicates. **** P < 0.001. **F**) Representative live-cell images of anti-CA9 ADC or isotype control ADC treatment (2.5 µg/ml) in normoxic and hypoxic U87MG cells at 96 h (scale bars, 450 µm).


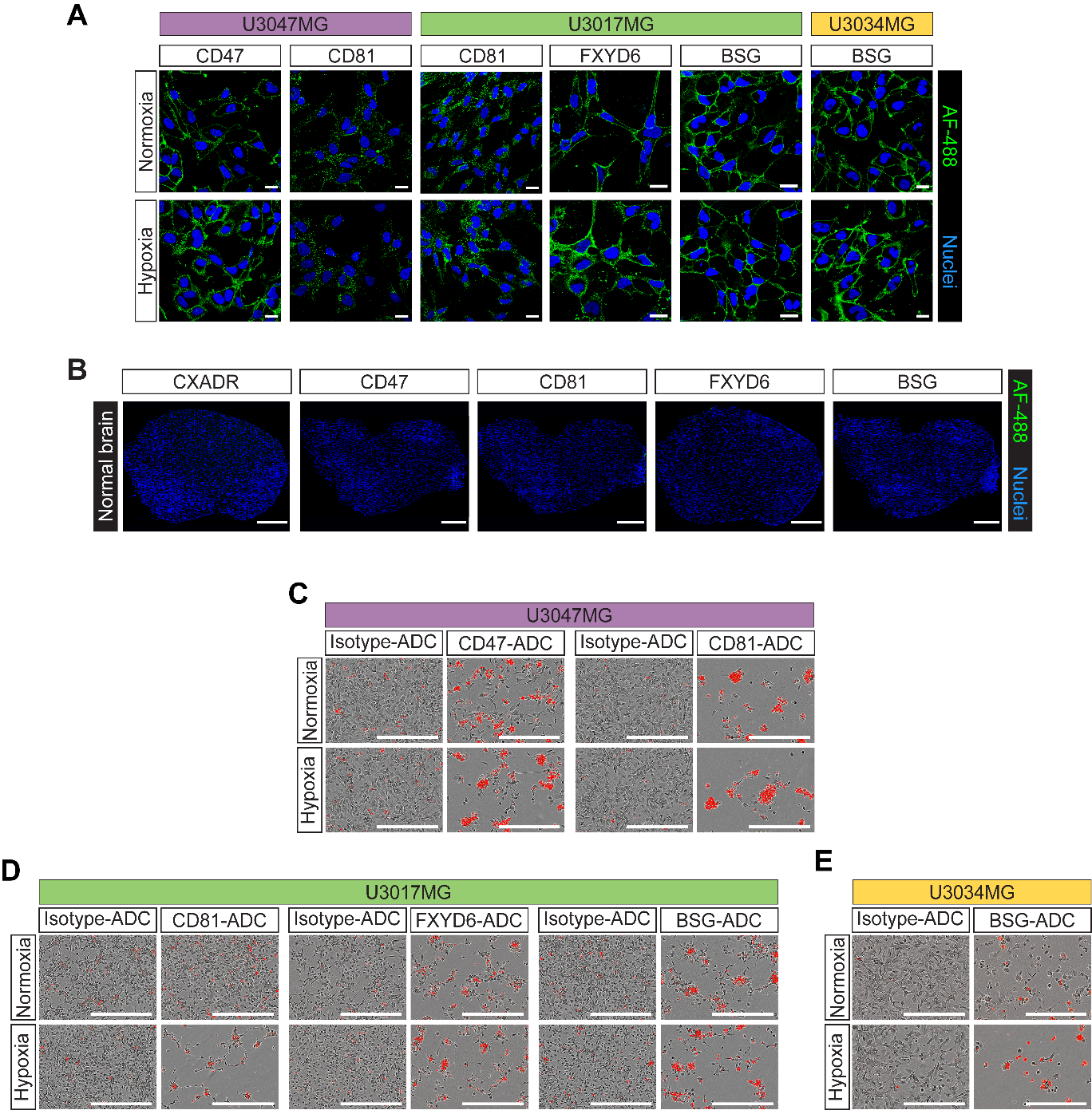


**Fig. S6. A)** Representative IF stainings for selected, hypoxia-induced targets, as indicated, in normoxic and hypoxic primary GBM 2D cultures, assessed by confocal microscopy (scale bars, 20 µm). **B**) Representative IF stainings for selected targets, as indicated, in human normal brain tissues from three separate individuals (scale bars, 1000 µm). **C-E)** Representative live-cell images at 96 h of ADC treatment directed at different targets or isotype control ADC, as indicated, in normoxic and hypoxic (**C**) U3047MG, (**D**) U3017MG, or (**E**) U3034MG cells (scale bars, 450 µm).
